# Short-term response to iron resupply in an iron-limited open ocean diatom reveals rapid decay of iron-responsive transcripts

**DOI:** 10.1371/journal.pone.0280827

**Published:** 2023-01-24

**Authors:** Joerg Behnke, Yun Cai, Hong Gu, Julie LaRoche

**Affiliations:** 1 Department of Biology, Dalhousie University, Halifax, Nova Scotia, Canada; 2 Department of Mathematics & Statistics, Dalhousie University, Halifax, Nova Scotia, Canada; University of Tasmania, AUSTRALIA

## Abstract

In large areas of the ocean, iron concentrations are insufficient to promote phytoplankton growth. Numerous studies have been conducted to characterize the effect of iron on algae and how algae cope with fluctuating iron concentrations. Fertilization experiments in low-iron areas resulted primarily in diatom-dominated algal blooms, leading to laboratory studies on diatoms comparing low- and high-iron conditions. Here, we focus on the short-term temporal response following iron addition to an iron-starved open ocean diatom, *Thalassiosira oceanica*. We employed the NanoString platform and analyzed a high-resolution time series on 54 transcripts encoding proteins involved in photosynthesis, N-linked glycosylation, iron transport, as well as transcription factors. Nine transcripts were iron-responsive, with an immediate response to the addition of iron. The fastest response observed was the decrease in transcript levels of proteins involved in iron uptake, followed by an increase in transcript levels of iron-containing enzymes and a simultaneous decrease in the transcript levels of their iron-free replacement enzymes. The transcription inhibitor actinomycin D was used to understand the underlying mechanisms of the decrease of the iron-responsive transcripts and to determine their half-lives. Here, Mn-superoxide dismutase (Mn-SOD), plastocyanin (PETE), ferredoxin (PETF) and cellular repressor of EA1-stimulated genes (CREGx2) revealed longer than average half-lives. Four iron-responsive transcripts showed statistically significant differences in their decay rates between the iron-recovery samples and the actD treatment. These differences suggest regulatory mechanisms influencing gene transcription and mRNA stability. Overall, our study contributes towards a detailed understanding of diatom cell biology in the context of iron fertilization response and provides important observations to assess oceanic diatom responses following sudden changes in iron concentrations.

## Introduction

As an essential trace nutrient, iron is scarce in the ocean’s surface water, and its complex chemistry results in multiple forms of iron with low bioavailability. Although free iron precipitates in the oxic contemporary ocean, over 99% of the iron is bound to organic chelators [1–3]. It is now well established that phytoplankton growth is iron-limited in 30–40% of the ocean’s surface waters [4,5]. Several iron fertilization experiments in low-iron areas resulted in diatom-dominated algal blooms, indicating the ability of diatoms to tolerate iron limitation and resume rapid growth once iron is resupplied [6–10]. Diatoms are cosmopolitan unicellular algae that build an essential part of the global marine food web and carbon export in the ocean [11]. Laboratory investigations of diatoms adapted to low-iron concentrations revealed several adaptations, including restructuring the photosynthetic apparatus, replacing iron-containing proteins, expressing high-affinity iron uptake systems, and increasing the surface/volume ratios [12–15]. While some adaptations in open ocean diatoms are permanent, many acclimations are transient and reverse upon iron resupply [12,14–17]. Overall, these adaptations substantially decrease the Fe: C ratio in open ocean diatoms [18]. In *Thalassiosira oceanica*, the reduction of the number of chloroplasts, the upregulation of endocytosis, and the upregulation of organic matter degrading enzymes are possible indications of a metabolic shift towards a mixotrophic lifestyle [14]. Diatoms are acclimated to the different chemical forms of iron by possessing and, often, combining a variety of uptake strategies, including a reductive iron uptake system [19], similar to the high-affinity uptake system found in yeast [20–22], iron uptake via a phytotransferrin, the iron starvation-induced protein 2 (ISIP2) [23], and an endocytosis-mediated siderophore uptake system via ISIP1 [24] and ferrichrome binding protein [25]. The response to changes in iron concentrations must be rapid as high intracellular concentrations of iron generate oxygen radicals toxic to the cell. Phytoplankton with a rapid response to iron resupply are adapted to physiologically acclimate to variable environmental conditions and can also dominate algal blooms after iron resupply events such as upwelling events [26], dust deposition [27,28] or volcanic eruptions [29]. In addition to natural events, anthropogenic iron fertilizations are events where phytoplankton communities experience sudden changes in iron concentrations [7].

In iron-limited cultures, transcript abundance for flavodoxin (FLDA) and ISIP3 in *T*. *oceanica* [30] and ferric reductases (FRE) in *Thalassiosira pseudonana* [31] were significantly downregulated within a few hours after the addition of iron. Proteomic analysis on the distantly related chlorarachniophyte *Bigelowiella natans* showed a rapid downregulation of flavodoxin and proteins involved in iron uptake within one hour after iron addition [32], suggesting that down-regulation and degradation are very rapid. However, these studies [30–32] lack a detailed temporal resolution of the changes in transcript abundance and only focus on specific genes. Here we were interested in investigating the exact time scale of the response to iron addition at the transcriptional and physiological level in the globally important open ocean diatom *T*. *oceanica*. The results support understanding temporal cell biological dynamics in unicellular phytoplankton in general and in the context of iron induction. Our study showed the downregulation of transcripts for iron uptake proteins as the primary response, followed by the upregulation of transcripts for iron-containing proteins that were replaced under iron limitation. Further, we analyzed mRNA transcript stability and found a half-life of under 12 min for all transcripts except Mn-superoxide dismutase (Mn-SOD) (90 min), cellular repressor of EA1-stimulated genes (CREGx2) (58 min), ferredoxin (PETF) (79 min) and plastocyanin (PETE) (67 min).

## Results

**Fig 1:**
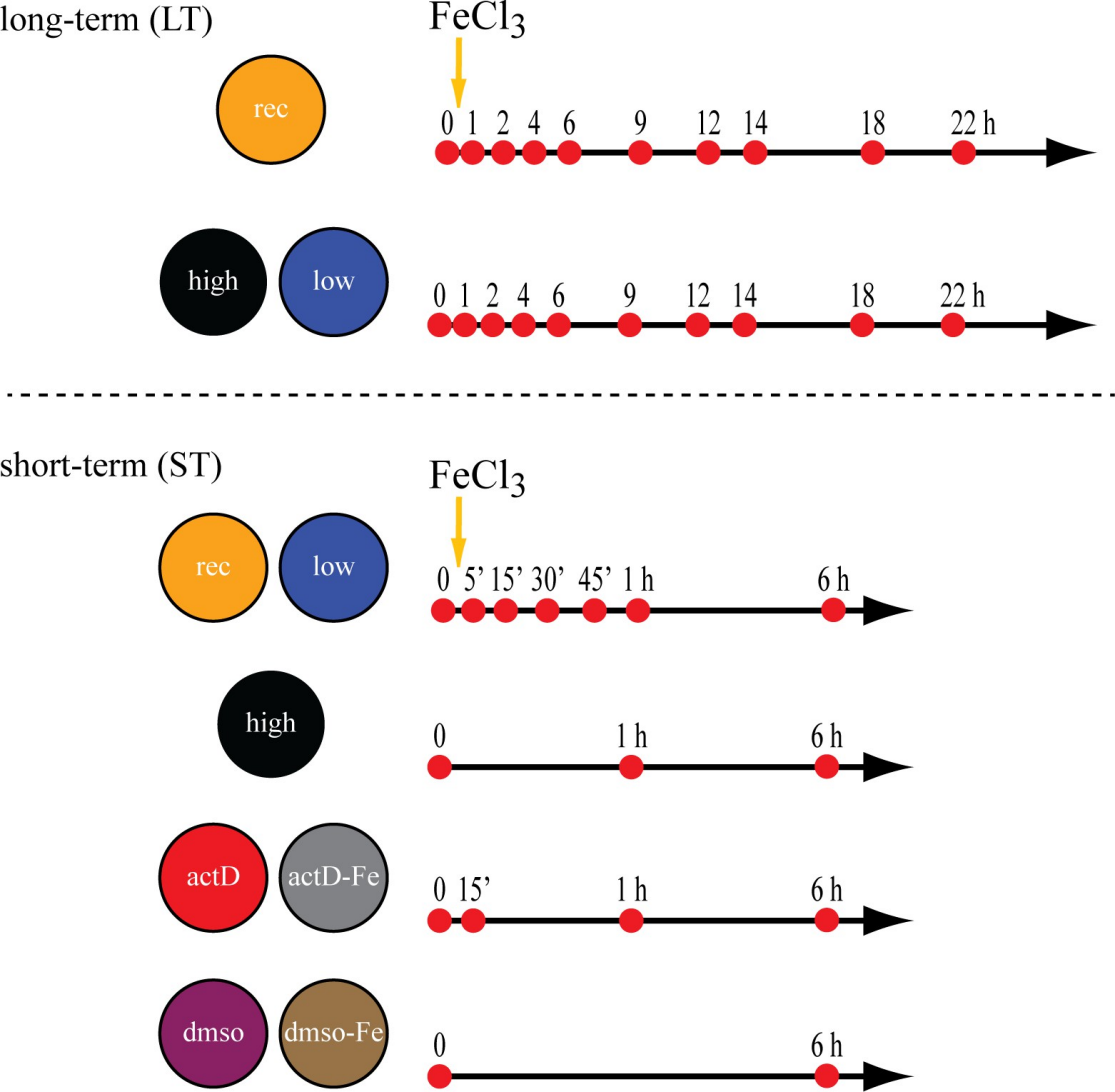
Overview of sampling times in the short-term and the long-term experiment. Overview of the sampling time points conducted during the long-term (LT) and the short-term (ST) experiment. The different treatments are indicated on the left, with the sampling times in hours (h) to the right. Orange arrows indicate the addition of iron with only the iron-recovery (rec), the actD-Fe and the dmso-Fe samples receiving 10 μM final concentration FeCl_3_ immediately after the initial measurement T_0_.

The effects of iron resupply to cultures of the open ocean diatom *T*. *oceanica* pre-adapted to iron-limited growth were analyzed at the transcriptional and physiological levels. The NanoString platform was used to track a total of 54 transcripts in two experiments. A first experiment was conducted following the response to iron resupply for 22 h (long-term experiment), demonstrating that some iron-responsive genes reached a new stable transcript level within the first hour. The second experiment, the short-term experiment (ST-experiment), was conducted over a 6 h period focusing on the first hour after iron addition. While 44 probes were designed for the first experiment (LT-experiment), 24 probes were designed to analyze all samples, the ST- and the LT-experiment (S2 Table). Both LT and ST experiments were conducted three times with different initial cell concentrations for the LT- and ST-experiment, respectively. The experiments included high-iron cultures grown with the addition of 10 μM FeCl_3_, low-iron cultures that were grown in filtered artificial seawater for trace metal removal without any addition of iron, and iron-recovery treatments where 10 μM final concentration FeCl_3_ was added after an initial (T_0_) measurement was taken. The additional treatments with Actinomycin D (actD) were used in the ST-experiment to inhibit transcription and analyze the transcript degradation rates. Here, Actinomycin D was added to low-iron treatments after the initial measurement was taken, either without the addition of iron or simultaneously with the addition of iron (Fig 1).

### Physiological response

**Fig 2:**
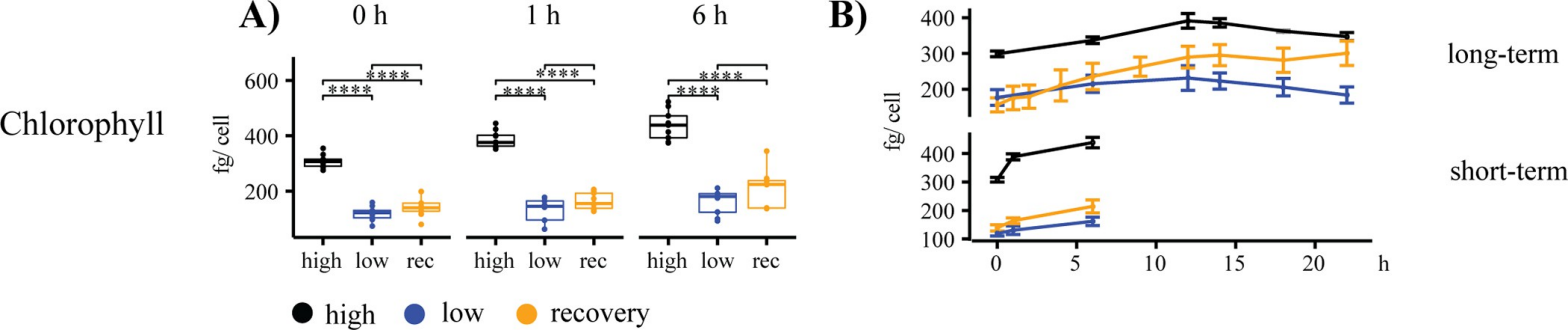
Changes in cellular chlorophyll content. Box plots (A) and short- and long-term time-courses (B) of chlorophyll. High-iron, low-iron, and iron-recovery samples are shown in black, blue, and orange, respectively. A two-tailed Student’s t-test was used for the statistical analysis of box plots (A). Box plots represent duplicate measurements of three experiments (n = 6) for each time point (0 h, 1 h, 6 h). Line graphs (B) show trends throughout the long-term and short-term experiments. Error bars represent standard errors (S.E.). Statistically significant P values are indicated as * <0.05, **, 0.01, *** < 0.001, **** < 0.0001. A line without stars indicates a test that is not statistically significant.

The temporal changes in chlorophyll revealed an increase in the chlorophyll content per cell for the iron-recovery samples (Fig 2). Flow cytometric measurements of fluorescence (FL3), side scatter (SSC) and forward scatter (FSC) were used as a proxy for cellular chlorophyll content, cell granularity, and cell size, respectively. The data of the ST- and the LT-experiment were combined and revealed significantly higher values for high-iron samples for all three parameters (S2 Fig). There were no significant differences between low- and high-iron samples in the chlorophyll content per cell after 6h, but high-iron values of FSC and FL3 were significantly higher than low-iron samples in the same time period (S1 Fig).

**Fig 3:**
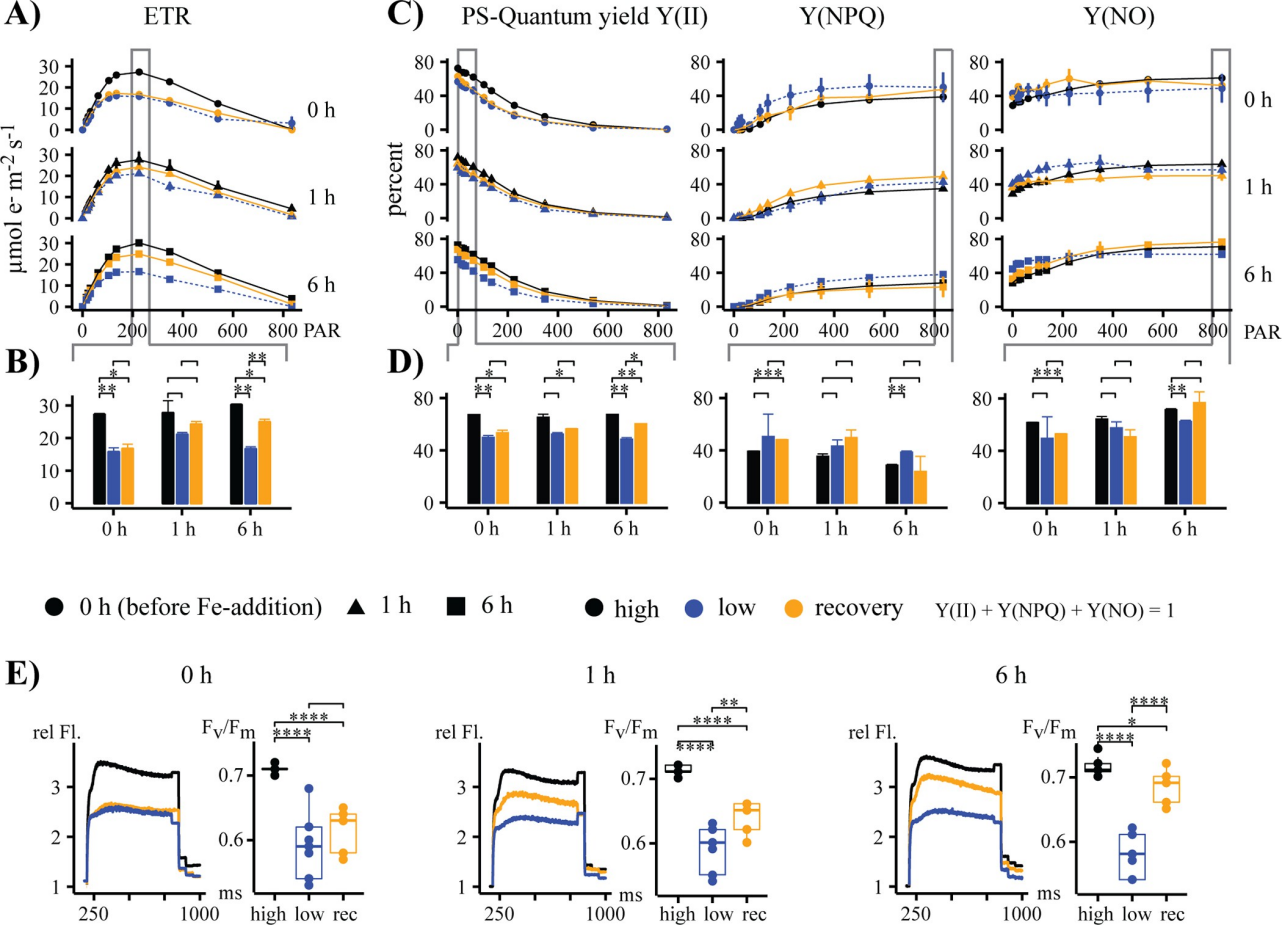
Changes in photo-physiological parameters throughout 6 h during the short-term (ST) experiment. High-iron, low-iron, and iron-recovery samples are shown in black, blue, and orange, respectively. The 0 h, 1 h, and 6 h sampling times are indicated as triangles, squares, and circles, respectively. (A) Electron transfer rate (ETR) in μmol e m^-2^s^-1^. (B) Bar graphs of ETR_max_ with Student’s t-test indicate statistically significant differences. (C) Photochemical quantum yield (PS-quantum yield Y(II)), non-photochemical quenching (Y(NPQ)), and non-regulated energy dissipation (Y(NO)). (D) Bar graphs of Y(II), Y(NPQ), and Y(NO) with Student’s t-test. (E) Light-induction curves and the corresponding F_v_/F_m_ values with ‘Student’s t-test showing statistical differences. Statistically significant P values are indicated as * <0.05, ** < 0.01, *** < 0.001, **** < 0.0001. A line without stars indicates a test that is not statistically significant.

High-iron cultures showed a faster electron transport rate (ETR) and higher photochemical quantum yield, Y(II), with iron-recovery samples approaching high-iron values within the first 6 h of the ST-experiment (Fig 3A–3D). Photochemical quenching, Y(NPQ), showed higher values in low-iron samples, while non-regulated energy dissipation, Y(NO), showed higher values in high-iron samples (Fig 3C and 3D). F_v_/F_m_ values and the corresponding light curves showed a significant upregulation after 6 h in the iron-recovery samples compared to low-iron samples (Fig 3E).

**Fig 4:**
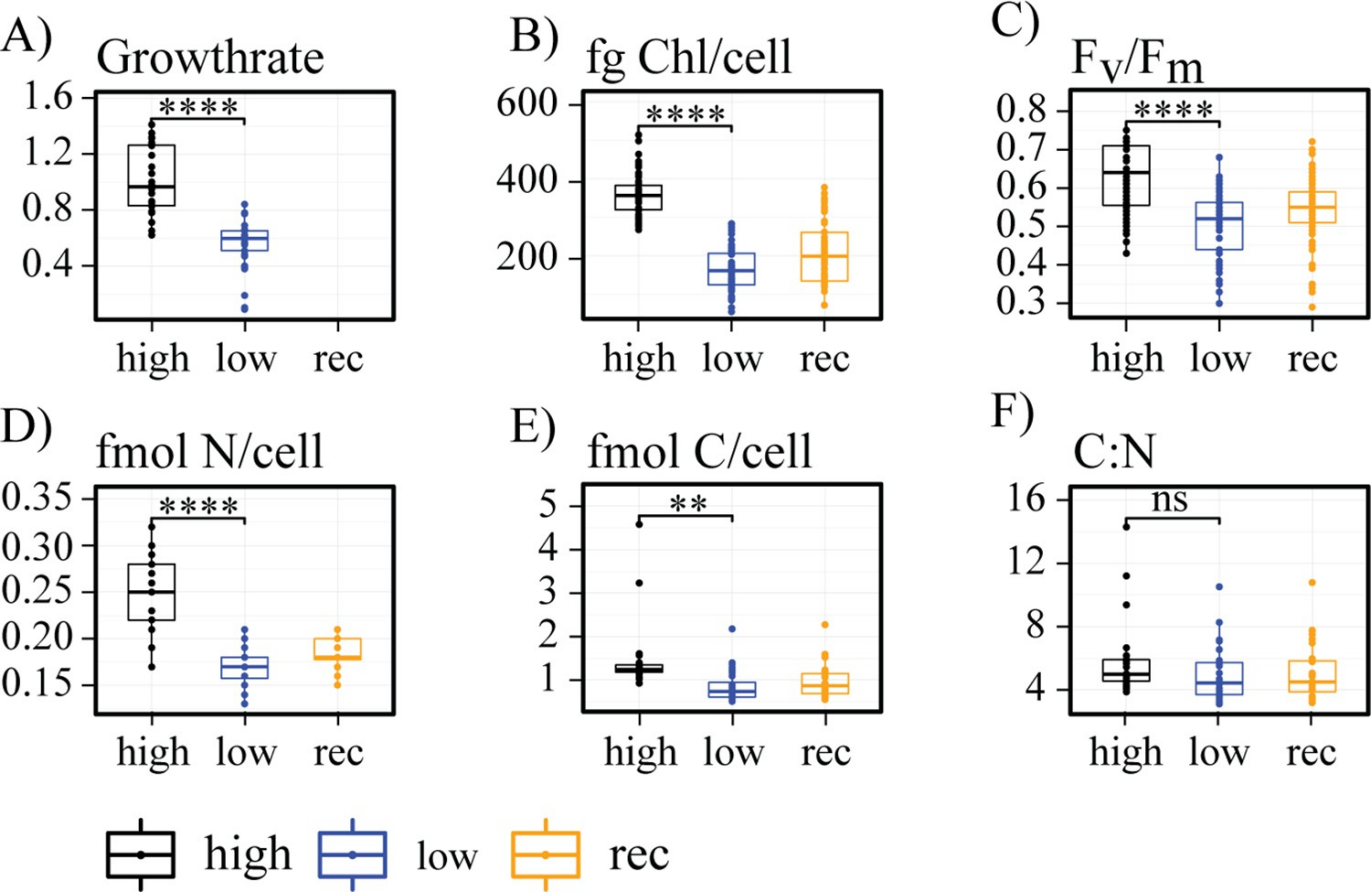
Comparison of physiological parameters in low-iron, high-iron, and iron-recovery samples. Characterization of cells grown under low-iron, high-iron, and iron-recovery conditions. High-iron samples are black, low-iron samples are blue, and iron-recovery samples are shown in orange. Results from ST- and the LT-experiment are combined, with every dot representing one sample. We used a two-tailed Student’s T-test for statistical analysis between high-iron and low-iron cultures. Statistically significant P values are indicated as * <0.05, ** < 0.01, *** < 0.001, **** < 0.0001. A line without stars indicates a test that is not statistically significant. (A) Growth rates μ [day^-1^]. (B) Chlorophyll content per cell (fg/cell). (C) F_v_/F_m_ measurements of all timepoints measured during LT- and ST-experiment. (D) Nitrogen/cell in fmol/cell. (E) Carbon/cell in fmol/cell. (F) C:N molar ratio.

Overall, acclimated (>1 year) low-iron and high-iron cells revealed faster growth and higher F_v_/F_m_ values for high-iron cultures (Fig 4A and 4C). Similar trends were observed in POC, PON and chlorophyll per cell with significantly higher values for high-iron treatments (Fig 4B, 4D and 4E).

### Dynamic response of transcript abundances for iron-responsive genes

**Fig 5:**
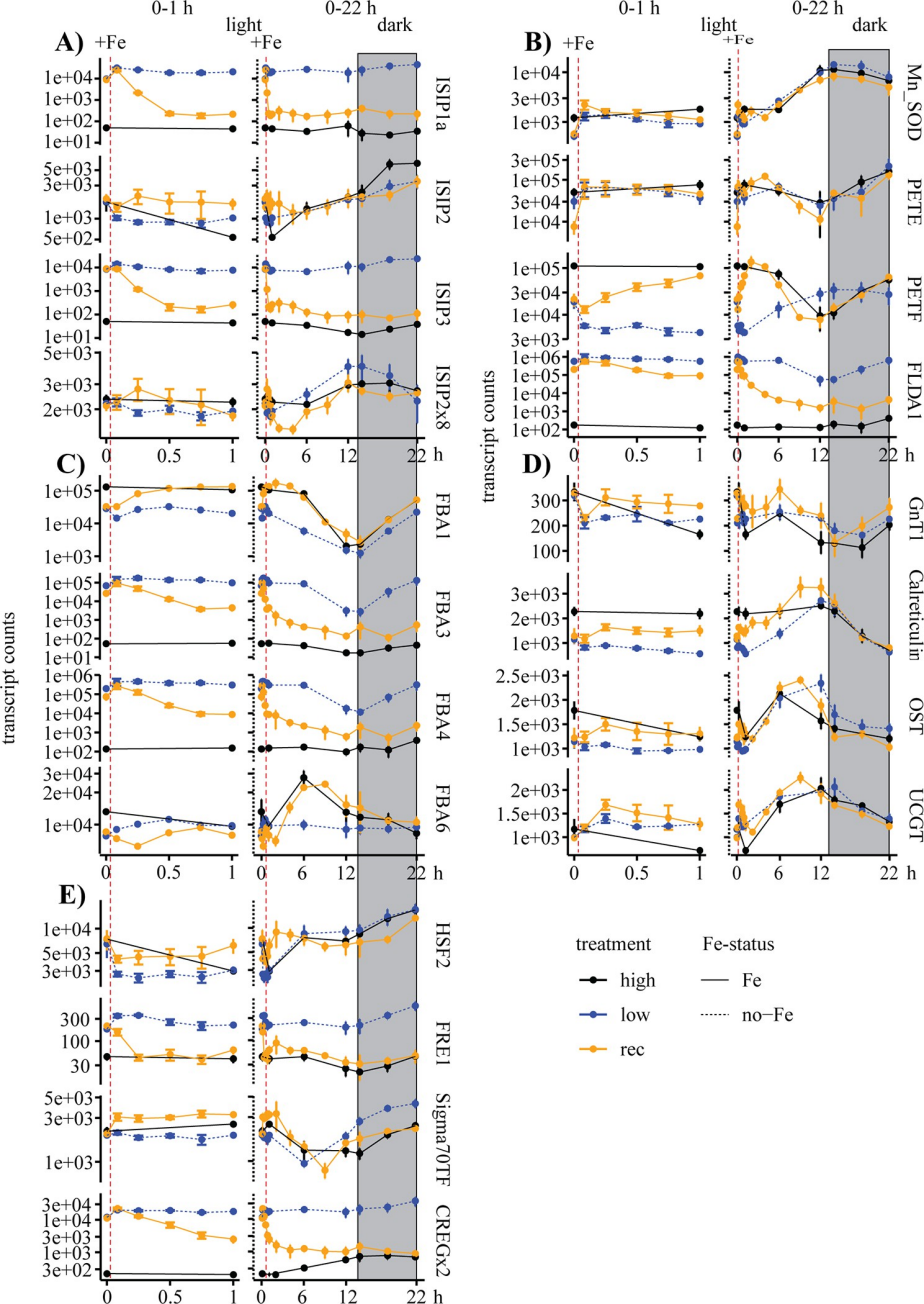
Time course of change in targeted transcript counts in high-iron, low-iron, and iron-recovery samples over the 6 h and the 22 h periods. Normalized transcript counts are on the y-axes vs time in hours on the x-axes, with 0 h representing 7 AM. Grey boxes indicate the dark phase. The response within the first hour is shown on the left, and the addition of iron is indicated with a red dashed line. Samples from the long-term experiment are shown on the right. High-iron, low-iron, and recovery samples are shown in black, blue, and orange, as in previous figures, with error bars indicating standard errors (S.E.). Transcripts are grouped: (A) iron starvation-induced proteins (ISIP), (B) proteins involved in photosynthesis, (C) Fructose-bisphosphate aldolases (FBA), (D) proteins involved in the glycosylation pathway, (E) transcription factors (HSF, Sigma70TF), iron reductase (FRE1) and a protein of unknown function (CREGx2).

The targeted transcripts were divided into five groups based on functional similarities (Fig 5). All four ISIP proteins (ISIP1, ISIP2, ISIP2x8, ISIP3) were combined in group 1 (Fig 5A), while transcripts involved in photosynthesis are in the second group (Fig 5B). More specifically, flavodoxin (FLDA1), superoxide dismutase (MnSOD), plastocyanin (PETE), and ferredoxin (PETF) are part of the second group. The fructose-bisphosphate aldolases (FBA1, FBA3, FBA4, and FBA6) form group 3 (Fig 5C). The other two groups are transcripts for proteins involved in glycosylation (group 4, Fig 5D), more specifically calreticulin, oligosaccharyltransferase (OST), N-acetylglucosaminyltransferase 1 (GnT1), and UDP-glucose glucosyltransferase (UGGT). Group 5 is comprised of 2 transcription factors (HSF2, SIGMA70), an iron reductase (FRE1) and CREGx2, a protein of unknown function (Fig 5E).

The temporal change in transcript levels following iron addition was the focal point of our study. Transcripts for iron-reductase, FRE1, showed the fastest acclimation to iron resupply and had declined to high-iron levels within 15 min. ISIP1 and ISIP3 transcript levels decreased to stable high-iron levels within 30 min upon iron addition. In contrast, ISIP2 and ISIP2x8 did not exhibit a well-defined response to iron. ISIP2 levels steadily increased throughout the 22 h period independently of the treatment, with a maximum in the dark period. Transcripts for PETF and FBA1 revealed a rapid increase, and while FBA1 reached high-iron counts after 30 min, PETF reached high-iron levels after 60 min. FLDA1, FBA3, and FBA4 showed an initial rapid decline but did not stabilize to high-iron levels within the 22 h period (Fig 5B and 5C). Similarly, CREGx2 showed a fast initial decline but reached high-iron levels after 12 h (Fig 5E).

Aldolases are essential enzymes in the Calvin cycle, glycolysis and gluconeogenesis, converting C3 and C6 sugars. The transcript levels differed based on their type and their predicted intracellular localization. FBA1 and FBA6 belong to the class-II aldolases with a divalent metal as a cofactor, whereas FBA3 and FBA4 are part of the class-I aldolases, bearing a Schiff-base as a cofactor. Within the acclimation response to iron-limitation, these proteins are part of the enzyme replacement strategy. FBA1 and FBA3 are both targeted to the chloroplast based on *in-silico* analysis. While the metal-containing FBA1 was upregulated following the addition of iron, the non-metal-containing FBA3 showed a rapid downregulation. Both transcripts showed a strong minimum at 7 PM and 9 PM for low-iron samples, with FBA1 exhibiting this trend in all three treatments and FBA3 showing very low counts for high-iron samples over all samples (Fig 5C). FBA4 and FBA6 are putative cytosolic proteins that showed differences in their transcript counts for all three treatments. In contrast, transcript levels for FBA3 and FBA4 were highly correlated (S7 Fig). In the iron-addition treatment, FBA6 followed the transcript counts of the high-iron samples, including a mid-day maximum that was absent in the low-iron samples (Fig 5C).

The chloroplast targeted Mn-SOD showed a clear pattern in all three treatments, with maximum counts between 9 PM and 1 AM. There was no response following the addition of iron, and all treatments followed the same trend. Similarly, PETE counts showed no differences between the three treatments, with a minimum expression before the dark period, followed by an increase. Following the addition of iron, flavodoxin transcript counts decreased rapidly (Fig 5B). Low-iron counts for FLDA1, FBA1, FBA3, and FBA4 showed a similar pattern with a minimum at the timepoints right before and after the start of the dark period (Fig 5B and 5C).

In order to analyze changes in the secretory pathway, four transcripts for enzymes involved in N-linked glycosylation were analyzed. High-iron and low-iron reads of these transcripts were included in a previous publication on N-linked glycosylation in microalgae [33]. The transcripts included OST, calreticulin, Gnt1, and UGGT. The only transcript with a response to the addition of iron was calreticulin, revealing an upregulation in the iron-recovery treatment during the light period compared to the low-iron samples (Fig 5D).

Group 5 was comprised of two iron-responsive genes, FRE1 and CREGx2, the heat shock factor HSF2 and the transcription factor SIGMA70 (Fig 5E). Counts of FRE1 and CREGx2 showed a fast decrease after iron addition. The heat shock factor HSF2 showed first a decline in the high-iron and iron-recovery treatments while the counts in the low-iron treatment increased. After six hours, all three treatments followed the same pattern, and a slight increase in counts was visible for all three treatments. The counts of the chloroplast targeted transcription factor SIGMA70 increased after the addition of iron, but the overall pattern appeared similar for all three treatments, with a minimum count around 6 h and a subsequent steady increase until the end of the dark period. Low-iron counts were elevated in the dark period compared to iron-recovery and high-iron samples (Fig 5E).

The counts of each transcript for both experiments, the ST- and the LT-experiment, were combined, and the fold change between low- and high-iron samples was calculated. The nine iron-responsive genes, including ISIP1, ISIP3, FRE1, FBA3, FBA4, FBA6, PETF, FLDA, and CREGx2 showed significant differences between the high- and low-iron samples. The highest fold change (3279x) was detected in FLDA1. FBA3 and FBA4 transcripts also revealed high fold changes (2142x and 1550x, respectively) (S3 Fig).

### Actinomycin D treatment decay curves

**Fig 6:**
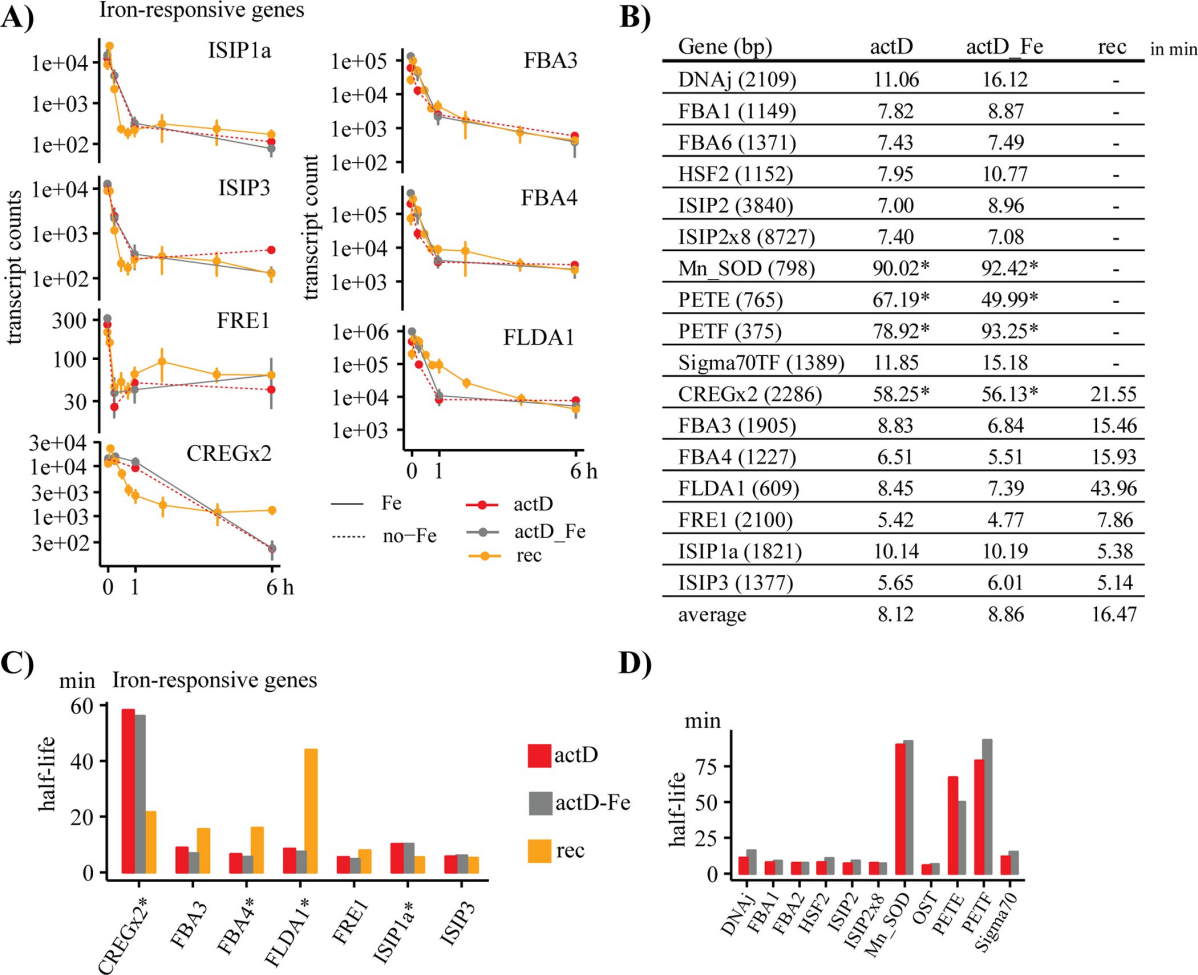
Overview of actinomycin D treatment on transcript counts compared to iron-induced reduction of mRNA transcripts. (A) Changes in transcript counts over time (6 h) in iron-recovery (rec; orange), actD-treated cells (actD–red, actD-Fe–grey). All samples shown here were low-iron samples. ActD cultures received actinomycin D and actD-Fe cultures received actinomycin D and iron. Rec are low-iron samples that received only iron. (B) Table of half-lives for transcripts analyzed in the ST-experiment (in min). Values with an asterisk are excluded from the average. (C) Bar graphs of half-lives of the 7 rapidly declining iron-responsive genes. Transcripts with an asterisk revealed significant differences between actD and iron-recovery samples. (D) Bar graphs of half-lives for non-iron-responsive genes.

To assess the stability of mRNAs under iron limitation, we used a transcription inhibitor, actinomycin D (actD). ActD was added only to low-iron samples, either in combination with iron (actd-Fe) or without iron (actD). Transcript half-lives were determined by comparing low-iron samples treated with and without the transcription inhibitor. Samples treated with actD showed a decrease in transcript levels for all targeted transcripts with differences in their respective half-lives (Fig 6). Four of the seven iron-responsive transcripts (ISIP1a, FLDA1, FBA4, and CREGx2) with a rapid decline after the addition of iron (Fig 6A) revealed significant differences in their decay rate comparing actD-treated samples with iron-recovery samples (Fig 6C).

The average half-life of all transcripts, excluding Mn-SOD, PETE, PETF, and CREGx2, was 8 min. The half-lives of Mn-SOD, PETE, PETF, and CREGx2 were between 50 and 90 min (Fig 6B). The actD experiment included the addition of actD to low-iron cultures as well as the addition of actD and iron to low-iron cultures. There were no significant differences between these two treatments for any targeted transcript (Fig 6C and 6D).

### Transcription factor analysis

**Fig 7:**
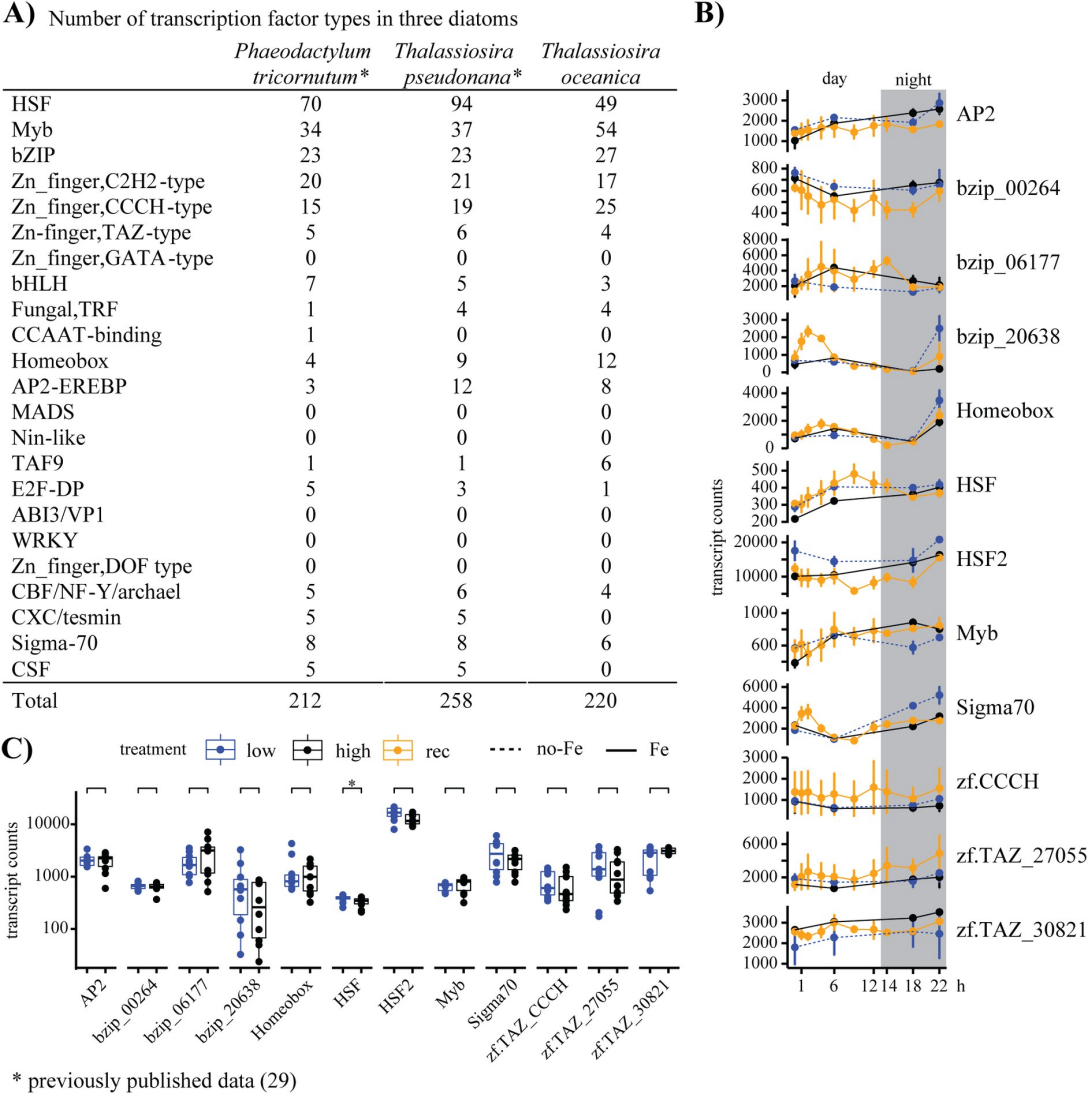
Putative transcription factors in *T*. *oceanica* and their transcript levels. (A) Comparison of previously published data on transcription factors in *P*. *tricornutum* and *T*. *pseudonana* [34] to *T*. *oceanica*. (B) Expression patterns of the transcription factors from high-iron (black), low-iron (blue), and iron-recovery (orange) treatments. Transcript counts are on the y-axes, and time (h) is on the x-axis. The grey box indicates the dark phase. (C) Transcript counts of high- (black) and low-iron (blue) treatments. A two-tailed Student’s t-test was used for statistical analysis. Transcript counts are on the y-axis, and the gene names are indicated on the x-axis.

The *in-silico* search for transcription factors (Tf) in the *T*. *oceanica* genome resulted in 220 putative Tfs with heat shock factors (HSF) and Myb Tfs accounting for around 50% of the Tfs (Fig 7A). A second large group included the bZIP proteins and Zn finger type Tfs (Fig 7A). Based on previous results [14], transcription factors differentially expressed under low- and high-iron conditions were analyzed in the targeted transcriptome analysis. Our data showed HSF2 as the only transcript with a significant difference between low- and high-iron samples (Fig 7C). Sigma70 and bzip_20638 showed an increase after the addition of iron. Therefore, Sigma70 was also included in the short-term analysis, and transcript counts increased transiently after the addition of iron (Fig 5E). However, the increase of Sigma70 and bzip_20638 after the first hour in the LT-experiment cannot be defined as iron-responsive as the control samples of high- and low-iron are missing for these timepoints (Fig 7B). Most transcription factors showed a steady expression throughout the 22 h, with a maximum at the end of the dark period for bzip_20638, Homeobox, HSF2, and SIGMA70 (Fig 7B).

### Changes in transcript levels following iron addition to iron-limited cultures

**Fig 8:**
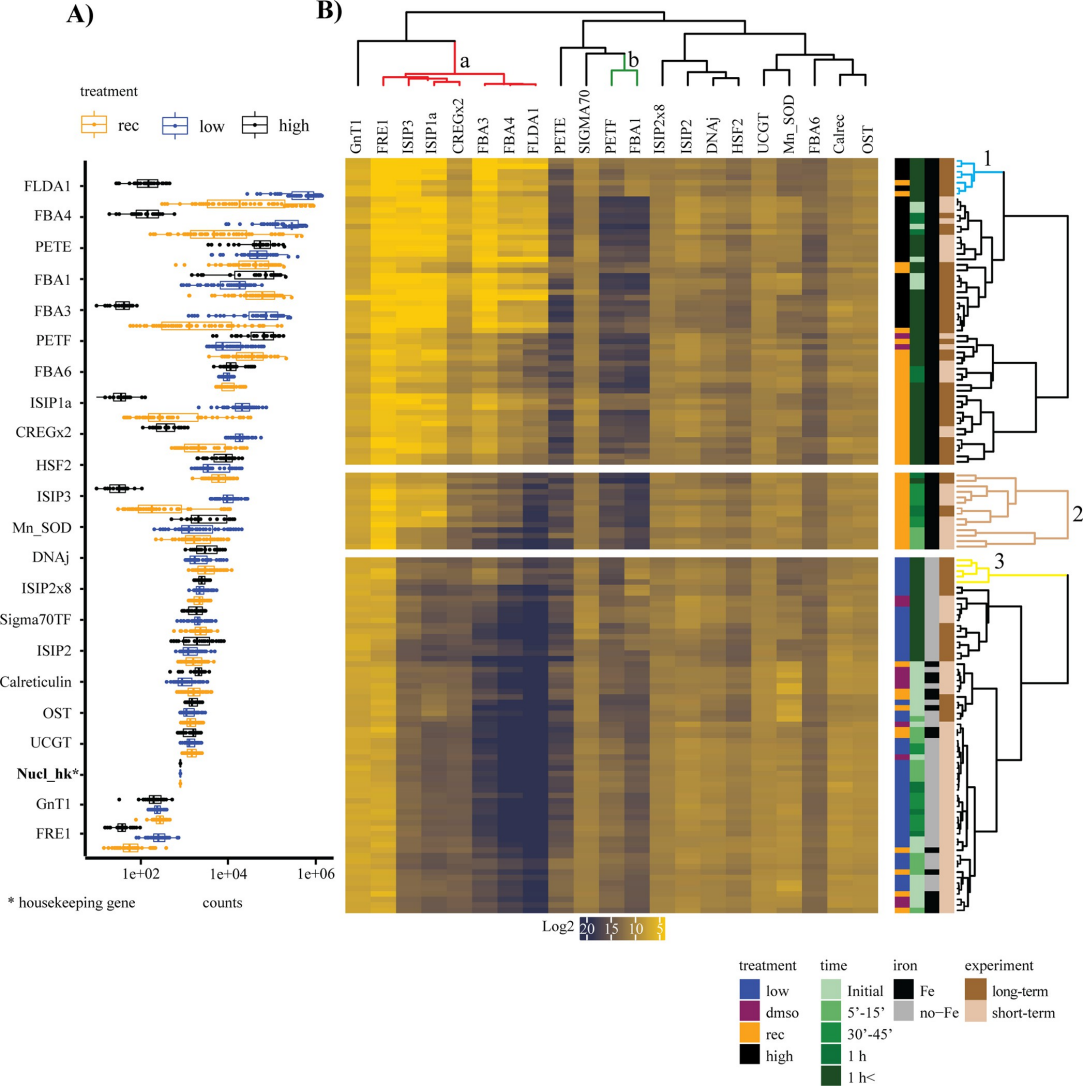
Normalized transcript counts for 21 targeted genes (A) and heatmap of Spearman correlation between transcript levels (B). (A) Normalized transcript counts (counts/100ng of total RNA). Low-iron (blue), high-iron (black), and iron-recovery (rec) (orange) samples are plotted for each gene. The x-axis is plotted in log-scale, with the genes plotted in order of decreasing transcript abundance. (B) Heatmap with clustering based on Spearman correlation. Transcript counts are plotted as log2 values, with yellow and dark blue indicating low and high counts, respectively. The four coloured bars represent treatments, sampling times, iron levels, and experiment type. For the treatment, low-iron samples are shown in blue, dimethyl sulfoxide (DMSO)-treated samples are purple, iron-recovery samples are shown in orange, and high-iron samples are black. Timepoints are separated into Initial, 5–15 min, 30–45 min, 1 h, and >1 h in increasingly darker shades of green, with darker shades indicating the time progression from earlier to later timepoints. Iron levels are indicated in grey for samples without iron and black for samples with iron. Samples from the long-term experiment are shown in dark brown, and samples from the short-term experiment are shown in light brown. The dendrogram above the heatmap indicates the following clusters: a) downregulated genes following iron addition and b) upregulated genes. The dendrogram on the left indicates clusters 1 and 3 with samples from 12 h and 14 h after iron addition. The split region shown as cluster 2 consists of iron-recovery samples from time points with rapid acclimation of the iron-responsive transcripts. The following abbreviations were used: flavodoxin (FLDA1), fructose-bisphosphate aldolase (FBA), plastocyanin (PETE), ferredoxin (PETF), iron starvation-induced protein (ISIP), cellular repressor of EA1-stimulated genes (CREG), heat shock factor (HSF), manganese-superoxide dismutase (Mn-SOD), transcription factor (TF), oligosaccharyltransferase (OST), nuclear import-export receptor house-keeping (nucl_hk), N-acetylglucosaminyltransferase (GnT1), UDP-glucose glucosyltransferase (UGGT), iron reductase (FRE1).

A Spearman correlation matrix of the above-described transcript levels of 21 genes revealed rapid changes after iron addition for nine genes (Fig 8). Transcript levels for iron reductase (FRE1), iron-starvation induced protein (ISIP) 1a, ISIP3, cellular-repressor-of-EA1-stimulated-genes x2 (CREGx2), fructose bisphosphate aldolase 3 (FBA3), FBA4, and flavodoxin (FLDA1) showed a sharp decline after the addition of iron (cluster a in Fig 8B). At the same time, FBA1 and ferredoxin (PETF) exhibited sharp increases in transcript levels following the addition of iron (cluster b in Fig 8B). The samples clustered predominantly based on the iron status creating a strict division between high- and low-iron samples (Fig 8B). Transcript levels for ISIP1, ISIP3, and FRE1 reached their minimum in the iron-recovery samples faster than the transcript levels for FBA3, FBA4, FLDA1, and CREGx2.

**Fig 9:**
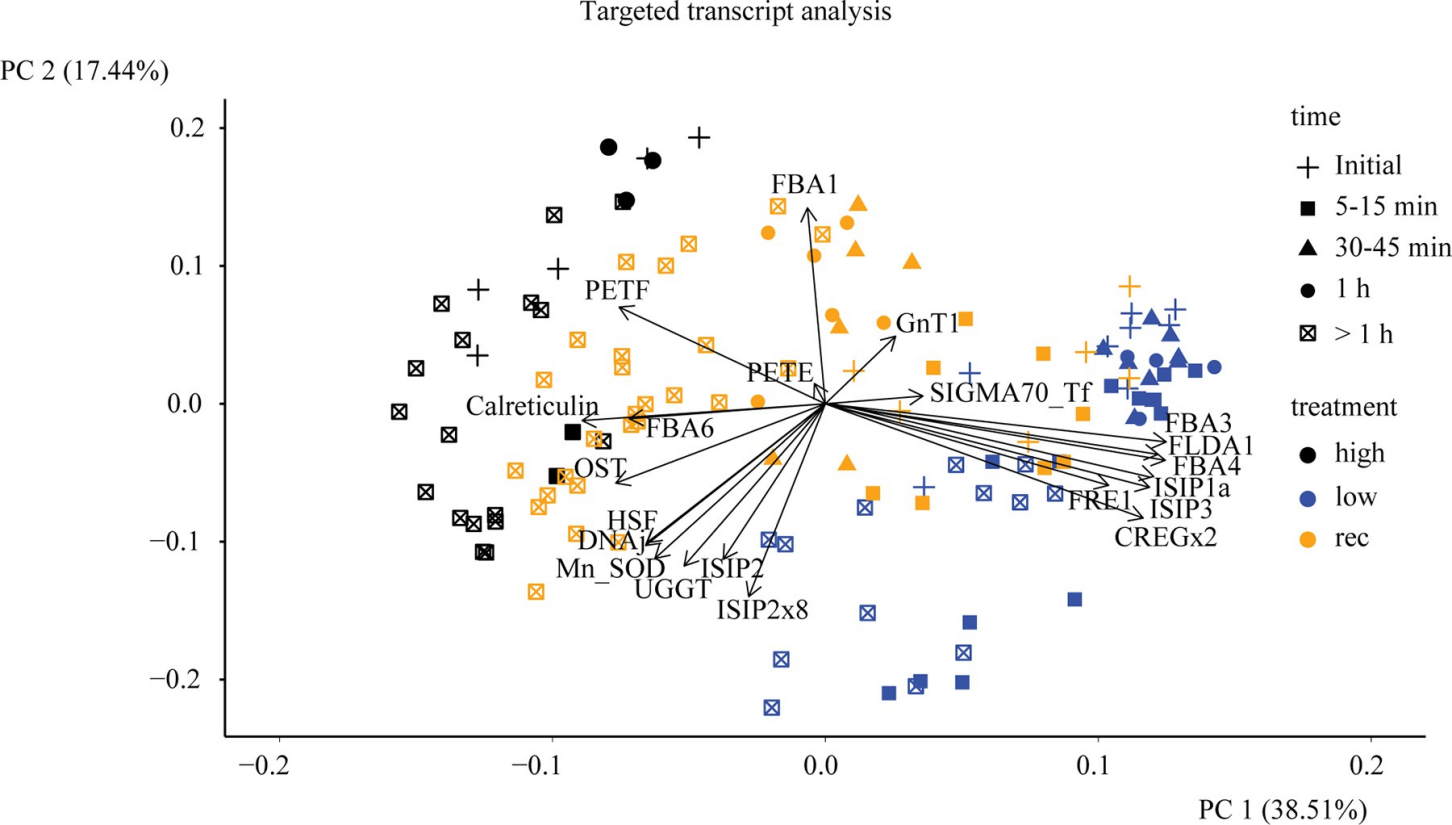
Principle component analysis. Principle component analysis (PCA) of all samples. Low-iron samples are shown in blue, high-iron in black and recovery samples are shown in orange. Sampling times are divided into groups and are shown as different shapes. The initial samples are represented by a plus sign, samples from 5–15 min are shown as a square, 30–45 min as a triangle, samples from one hour are shown as a dot, and all samples after one hour are shown as a crossed square.

A principle component analysis (PCA) revealed the same trend and showed a strict division of the samples based on their iron status and, for iron-recovery samples, a division based on the sampling time (Fig 9).

## Discussion

The cellular response of phytoplankton to iron limitation has been a focus of research efforts since the early 1990s, soon after in-situ iron fertilization experiments demonstrated prompt algae growth upon iron addition resulting in diatom-dominated blooms [7,9]. Laboratory studies on specific diatom species followed, revealing significant differences between high- and low-iron acclimated diatoms at the transcriptional and physiological levels [14,16,20,31,35,36]. We analyzed the short-term response to iron resupply in the globally important open ocean diatom *T*. *oceanica*. This oceanic diatom is well adapted to low-iron concentrations, including changes in the stoichiometry of photosynthetic proteins, the upregulation of proteins involved in iron uptake systems, and the replacement of iron-containing proteins with non-iron-containing proteins [14,37,38]. While most studies have focused on adaptive strategies to survive chronic iron limitation, our experiments have focused on the short-term response to iron resupply in iron-limited diatoms.

### Changes in transcripts for iron uptake-related proteins

A sudden increase in extracellular iron concentrations can potentially lead to damaging intracellular iron concentrations. Therefore, adjustments in iron uptake must be fast, and our data showed a rapid decrease of transcripts for iron uptake-related proteins. *T*. *oceanica* possesses several proteins involved in iron uptake that are strongly upregulated in low-iron conditions, including ISIP1, ISIP2, iron reductases, and multicopper oxidases [14,17,35,36,39,40]. The classic high-affinity uptake system is a reductive uptake system involving an iron reductase [41]. Ferric-reductase (FRE) activity increased under iron-limitation in *Phaeodactylum tricornutum* [36], and reduction plays a role in capturing organically bound iron in *P*. *tricornutum* [25] and *T*. *oceanica* [21]. Our data confirmed the upregulation of the iron reductase FRE1 under low-iron conditions, and FRE1 transcript levels decreased to high-iron levels within 15 min (Fig 6E). The overall low counts of FRE1 may indicate minor importance of the reductase activity in *T*. *oceanica* or high enzymatic efficiency. Diatoms evolved different iron uptake systems, with ISIP1 and ISIP2 involved in two different iron uptake systems [23,24,42]. Although the role of ISIP1 is not fully understood, it is likely part of an endocytosis-mediated uptake system for iron-siderophore complexes, delivering iron to the chloroplast [24]. In contrast, ISIP2 is a general ferric ion uptake protein belonging to the transferrin protein family, requiring carbonate ions for its functionality [23]. We analyzed all four ISIPs (ISIP1, ISIP2, ISIP2x8, and ISIP3) present in *T*. *oceanica* to uncover their immediate response to iron resupply. Surprisingly, only ISIP1 and ISIP3 showed a rapid downregulation after the addition of iron. Transcripts of both ISIPs stabilized at high-iron levels within 30 min (Fig 5A). The expression patterns of ISIP1 and ISIP3 showed a high correlation (R^2^ = 0.94) (S6 Fig), which suggests a coordinated regulation and common function. ISIP3_*To*, heterologously transformed into *P*. *tricornutum*, showed a similar localization to siderophores internalized through ISIP1 [19,24]. We hypothesize that ISIP1 and ISIP3 are likely functionally linked and involved in the iron homeostasis of the chloroplasts, but the exact role of ISIP1 and ISIP3 still needs to be shown. While ISIP1 and ISIP3 showed a strong response to the addition of iron, ISIP2 and ISIP2x8 revealed no changes in their transcript levels after the addition of iron (Fig 5A). This is in contrast to previous studies that showed a strong upregulation of ISIP2 under low-iron conditions in *T*. *oceanica* [14,36]. More detailed molecular work showed that ISIP2 functions as a phytotransferrin with a co-dependence on carbonate ions [23]. With all carbonate binding sites present in *T*. *oceanica* [19], carbonate concentration in our media could have influenced the expression of ISIP2 genes.

### Cellular-repressor-of-EA1-stimulated-genes x2 (CREGx2)

**Fig 10:**
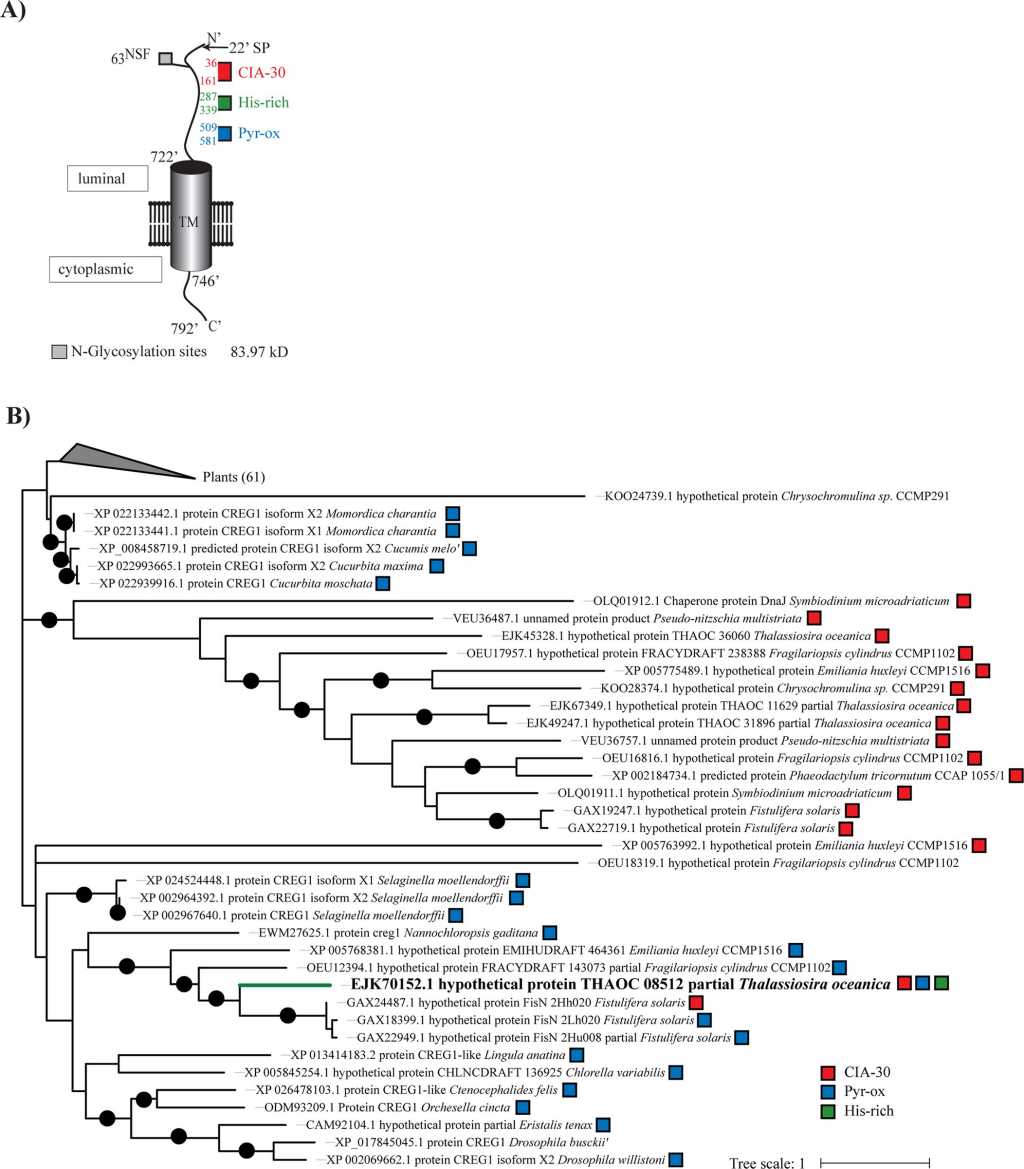
Structure and evolutionary distance to other CREG proteins of CREGx2_*To*. (A) CREGx2_*To* protein structure. Transmembrane domain (TM) and N-glycosylation sites are indicated as barrels and squares, respectively. (B) Phylogenetic tree of CREG proteins. The *T*. *oceanica* sequence is shown in bold (THAOC08512). The maximum likelihood tree was generated in MEGA [44] using the top 100 blastp hits. The sequence alignment is based on a muscle alignment, and the tree was generated with the WAG model and gamma distribution. The black circle represents a bootstrap of > 0.5. Three conserved domains are indicated. Complex I intermediate-associated protein 30 (CIA-30) is in red, Pyrodoxamin-5’phosphate oxidase (Pyr-ox) in blue and histidine-rich region (His-rich) is indicated in green. The signal peptide is abbreviated with SP.

The CREGx2 protein was further analyzed *in-silico* based on its strong upregulation under low-iron conditions (S3 Fig) and its immediate response to iron addition (Fig 5E). CREG proteins were identified as an iron-responsive gene in *P*. *tricornutum* [36], *T*. *oceanica* [14], and *B*. *natans* [32], upregulated under iron limitation. It was one of the fastest downregulated proteins after iron addition in *B*. *natans*, reaching high-iron levels within 24 h [32], and it was found in close proximity to phytotransferrin (ISIP2a) in *P*. *tricornutum* [43]. *In-silico* analysis of CREG proteins in *P*. *tricornutum* and *B*. *natans* resulted in suggested functions as a phytotransferrin-controlling protein [43] and a growth-controlling protein [32], respectively. The *in-silico* analysis on CREGx2 in *T*. *oceanica* points towards a different function. We found a complex I intermediate-associated protein 30 (CIA30) domain, a pyridoxamine 5’-phosphate oxidase (P5P) domain and, most interestingly, a histidine-rich site in CREGx2_*To*. Structurally, CREGx2_*To* contains a transmembrane domain and a signal peptide, predicting CREGx2_*To* to enter the secretory pathway (Fig 10A). A phylogenetic tree of the TOP100 hits of a blastp search showed that CREGx2_*To* clustered in a small clade differentiating CREGx2_*To* from other CREG proteins that contain only one of the conserved domains (Fig 10C).

His-rich regions are typically found in ZIP proteins and functionally relevant for divalent metal transport [19,45]. Our analysis shows CREGx2_*To* as the only protein with all three identified domains and the only protein with a His-rich region. Our analysis led to the hypothesis that CREGx2_*To* is involved in iron homeostasis, based on the strong upregulation under iron limitation, the immediate response to iron addition, and, most importantly, the histidine-rich site pointing towards iron-binding. The actD analysis revealed a significantly longer half-life of the transcripts after the addition of iron than in the actD treatment, indicating that regulatory processes may reduce mRNA degradation of CREGx2_*To* (Fig 6).

### Response of the photosynthetic apparatus

Iron limitation leads to reduced chlorophyll content and a general downregulation of chloroplast transcripts in *T*.*oceanica* [14]. Our study revealed a fast recovery of photo-physiological parameters, including ETR, Y(II), and F_v_/F_m_. All three parameters showed a strong recovery within the ST-experiment, indicating a fast improvement of the photosynthetic apparatus (Fig 3). These findings align with transcriptomic field studies on diatoms from the genera *Pseudo-nitzschia* and *Thalassiosira sp*., where iron addition resulted in an upregulation of genes involved in the photosynthetic apparatus within 48 h [46]. In our study, F_v_/F_m_ increased within 1 h and reached almost high-iron values at the 6 h time point, which was faster than the recovery of the photosystem II in *B*. *natans* [32], where more than 24 h was needed. Structural changes within the PSII complex, as observed in *Chaetoceros simplex* [47], and the increase in F_v_/F_m_ may have resulted from an increase in PSII-D1 protein content [14]. While F_v_/F_m_ displayed a fast recovery in our study, the chlorophyll content per cell (fg/cell) did not reach high-iron levels within the 22 h time frame of our study (Figs 2 and 3E).

FLDA1 replaces ferredoxin as a terminal electron acceptor in the photosynthetic apparatus channelling electrons from photosystem I to the NADH-reductase. It was described as an iron-regulated protein in algae in the mid-1990s and further suggested as an indicator for iron limitation in phytoplankton [12,30,38,48]. The use as an indicator has been successful [49] but is complicated as some diatom species express FLDA constitutively [50], FLDA expression can be reduced under conditions of iron and manganese co-limitation [51], and a transient alleviation of iron limitation can lead to an upregulation of FLDA1 [40,52]. FLDA proteins have been categorized into iron-responsive and iron-neutral types [53], with FLDA1_*To* belonging to the iron-responsive type [14,53]. In our study, FLDA1 showed the highest fold change between high- and low-iron samples (S3 Fig), but the transcript levels after the addition of iron remained higher than the transcript levels we found in the high-iron treatment (Fig 5B). The expression of FLDA1 is foremost to replace PETF, which is present when iron concentrations are sufficient. Despite the slow decrease in FLDA1 transcript levels, transcript levels for PETF increased rapidly and reached high-iron levels within 1 h. Besides replacing FLDA1, the fast upregulation of PETF could result from the upregulation of ferredoxin-dependent enzymes such as glutamate synthases and sulfite reductases, as seen in field samples for the genera *Thalassiosira* [46]. PETF had ten times lower transcript counts in our analysis than FLDA1, suggesting a lower efficiency of FLDA1 or a broader range of functions for FLDA1 under iron limitation (Fig 5B). We also detected a half-life for PETF of 78 min, which is much longer than the calculated average half-life, indicating a constant demand for PETF in the cell (Fig 6B).

In contrast, the half-life of FLDA1 in the actD treated samples was only 8 min, with a significant difference between the decrease in transcript levels after iron addition and the decrease after transcription inhibition through actD (Fig 6B). This difference points towards a regulatory transcript controlling the transcription itself or the transcript stability of FLDA1 as the regulatory transcript would not be transcribed in the actD samples.

Plastocyanin (PETE), a replacement protein for cytochrome c_6_, is mainly found in low-iron adapted diatoms. PETE possesses copper as a cofactor and is therefore regulated by copper availability [54]. Based on the low expression of cytochrome c_6_ in *T*. *oceanica* cultures in high- and low-iron conditions, it has been suggested that PETE replaces cytochrome c_6_ permanently [14]. This theory was further supported by studies in the North Pacific, where a permanent replacement of cytochrome c_6_ in low-iron adapted diatoms and a temporary replacement of cytochrome c_6_ in coastal diatoms was suggested. In contrast to previous findings [14], our study did not show differences in PETE transcript levels between the three treatments. These differences could stem from a lower iron concentration in the previous study [14]. However, the long half-life supports the hypothesis of a permanent replacement of cytochrome c_6_ by PETE in *T*. *oceanica* (Figs 5B and 6D) [13–15].

### Aldolases enzyme replacements

Aldolases are critical enzymes in glycolysis, gluconeogenesis, and the Calvin-Benson cycle catalyzing the conversion of fructose-1,6- bisphosphate (FBP) into dihydroxyacetone phosphate (DHAP) and glyceraldehyde-3-phosphate (GAP). The gene expression is according to their cofactor and varies based on the iron status [46]. We analyzed four FBA proteins, FBA1, 3, 4, and 6, in our targeted transcriptome analysis (Fig 5C). While FBA1 and FBA6 belong to the class-II aldolases and require a divalent metal in their active site, FBA3 and FBA4 belong to the class-I aldolases using a Schiff-base cofactor. Based on the involvement of aldolases in various processes, FBA proteins in *P*. *tricornutum* are present in the cytosol, in the chloroplast and pyrenoids [55]. Two FBA enzymes in *T*. *oceanica*, FBA1 and FBA3, are localized in the pyrenoids [14]. Our *in-silico* analysis supported that FBA1 and FBA3 are targeted to the chloroplasts, while FBA4 and FBA6 are cytosolic. The two class-II proteins, FBA3 and FBA4, had, together with FLDA1, the greatest fold change between high- and low-iron samples (S3 Fig), and transcript levels decreased immediately after iron addition (Fig 5C). The expression data of FBA3 and FBA4 correlated very strongly (R^2^>0.97), but cross-hybridization can be excluded based on the probe sequence alignments that indicated identities of under 75% [56] (S7 Fig). The strong correlation was unexpected, considering the differences in their predicted localization, the resulting putative function, and a complete phylogenetic separation [55]. An *in-silico* search for regulatory elements upstream of FBA3 and FBA4 did not result in any common putative promoter sequences, and the previously identified iron-responsive promoter is only present in FBA3 [14,57]. Both transcripts had longer half-lives in the iron-recovery samples compared to the actD samples, which could result from the transcription of a regulatory element that influences transcription or mRNA stability, similar to FLDA1. Transcript levels in low-iron treatments of FBA3 and FBA4 and all treatments of FBA1 followed a diel cycle similar to enzymes of the Calvin-Benson in *P*. *tricornutum* [35]. Based on their localization and their type, FBA1 and FBA3 could be each other replacing enzymes, but the low fold change (log2 = -1.97) of FBA1 and its expression in the low-iron samples itself showed that FBA1 was still transcribed under iron-limiting conditions and not entirely replaced by FBA3. In contrast to FBA1 and FBA3, expression patterns of FBA6 and FBA4 are very different, indicating different functions in the cell (Fig 5C).

### Transcription factor analysis

The results of the *in-silico* analysis of the *T*. *oceanica* genome for transcription factors (Tf) showed similar numbers of specific types compared to *P*. *tricornutum* and *Thalassiosira pseudonana* [34] (Fig 7A), suggesting that genome size does not appear to correlate with the number of transcription factors. The genome of *T*. *oceanica* is with 80 Mbp more than double the size of the genome of *T*. *pseudonana* (32 Mbp) and *P*. *tricornutum* (27 Mbp) [19]. However, these observations may result from the less complete assembly of the *T*. *oceanica* genome, which consists of over 50,000 scaffolds [14]. The long-term experiment included the analysis of 12 transcription factors, with only one transcription factor from the heat shock factor family showing a significant difference between high and low-iron samples (Fig 7C). The chloroplast-targeted transcription factor SIGMA70 is one of three transcription factors upregulated under iron limitation and implicated in the control of the chloroplast genome in *P*. *tricornutum* [35]. One of the SIGMA70 transcription factors in *T*. *oceanica* was putatively targeted to the chloroplast and was transiently upregulated under iron limitation in the LT-experiment (Fig 7C). Its immediate increase in transcript levels after iron induction seems contrary to the overall downregulation, but measurements for high- and low-iron samples are missing for these timepoints, limiting the interpretation of our results. Overall, we did not detect strong significant differences between high- and low-iron samples for any of the transcription factors analyzed, and the response to iron addition was not well defined (Fig 7).

**Fig 11:**
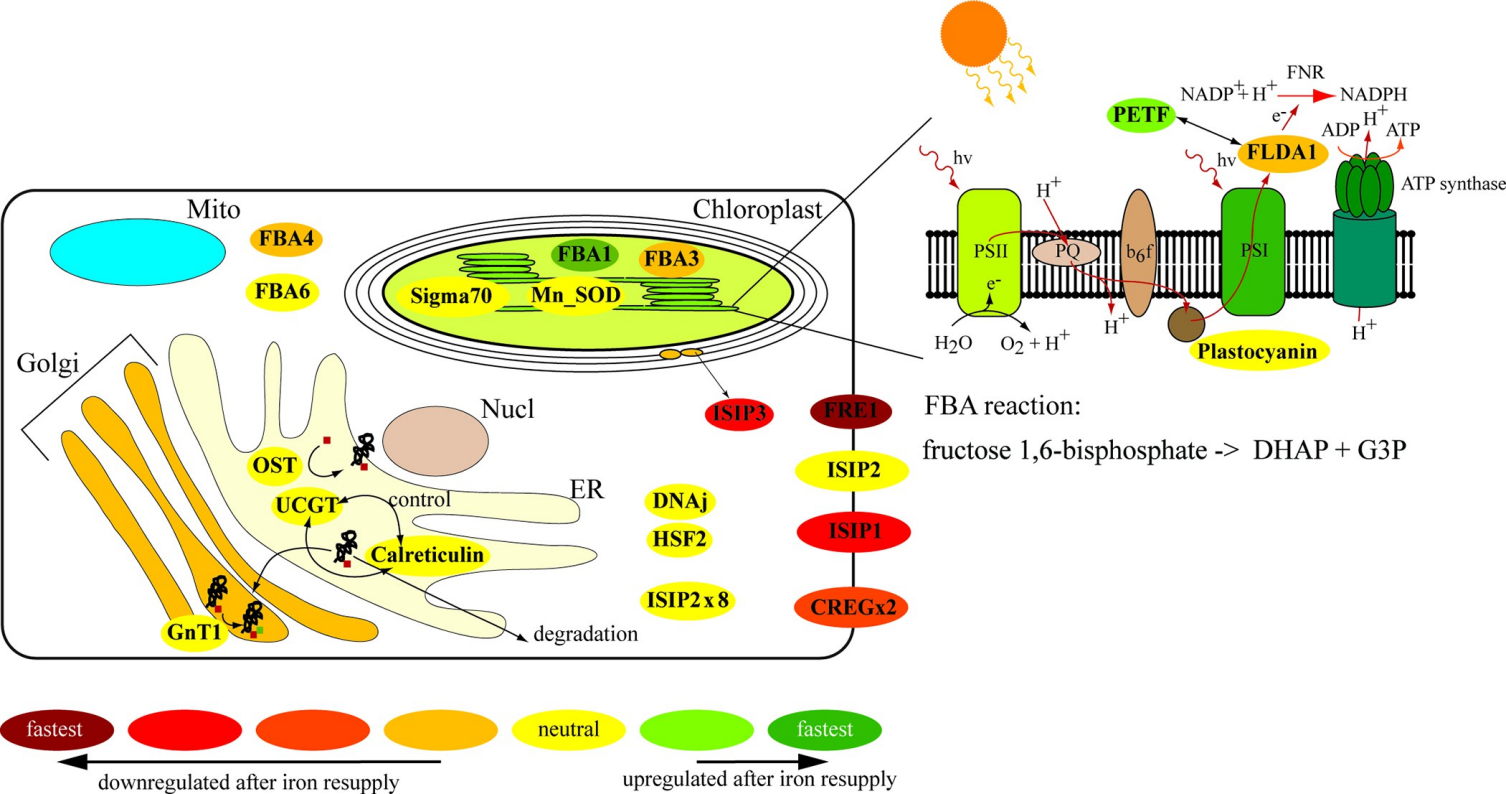
Cellular overview of the targeted transcripts and their localization in the cell. The scheme of a *T*. *oceanica* cell is shown with chloroplast (light-green), mitochondrion (Mito/blue), endoplasmic reticulum (ER/beige), Golgi apparatus (Golgi/orange), and the nucleus (Nucl/brown) indicated. The position of the gene name indicates the localization with respect to published data (ISIP1, ISIP2, ISIP3, FBA1, and FBA3) or putative location based on a prediction by TargetP [58] and ChloroP [59]. The surrounding colour shows the state and the temporal change of regulation upon iron resupply with upregulated genes shown in orange, dark orange, light red, and dark red indicating slow to fast acclimation, respectively. The downregulated genes are shown in two shades of green with the darker green indicating the fastest acclimation, and neutral genes are shown in yellow.

In conclusion, our data showed a high-resolution timeline of the events following a resupply in iron (Fig 5), analyzing transcripts of proteins from various locations in the cell (Fig 11). The cell’s survival depends on the balance between iron supply and demand while minimizing the toxic effect of free intracellular iron ions. We showed that proteins involved in iron uptake that are upregulated under low-iron conditions, including ISIP1, ISIP3, FRE1, and CREGx2, were downregulated first, limiting excess import of iron into the cell. Except for CREGx2, these transcripts were fully downregulated within 30 min after the addition of iron (Fig 5A and 5E). Further, our data revealed that transcripts of iron-containing proteins that were replaced by non-iron-containing proteins under low-iron conditions were immediately upregulated, increasing the demand for iron and buffering the increased influx of iron. Their iron-free counterparts were downregulated after the addition of iron. Most of these changes started immediately after the addition of iron, but when compared to the transcripts of iron uptake proteins, their complete downregulation was slower (Fig 5B and 5C). These immediate changes improved photo-physiological and cellular parameters such as cell size, photochemical quantum yield, electron transfer rate, chlorophyll content and Fv/Fm. All values increased significantly within the first six hours after the addition of iron (Figs 2, 3, S1 and S2). The ability of *T*. *oceanica* as an open ocean diatom to rapidly adjust its transcriptome in response to a sudden increase in iron concentration explains the success of open ocean diatoms in iron-induced algae blooms and provides novel insight into the plasticity of their cell biology.

## Material and methods

### Artificial seawater and culturing

Axenic cultures of *Thalassiosira oceanica* (CCMP1005) were grown in ASW f/2 with a 14/10 h light/dark cycle at 22°C using trace metal clean polycarbonate (PC) bottles. ASW was prepared after Goldman et al. [60] with nitrate (NO_3_) as the only nitrogen source. The Aquil metal mix [61] was used without Ni^2+^ using a 100 μM EDTA final concentration. High- and low-iron water was prepared in a trace-metal clean fashion, including all equipment used for the experiment. Stock cultures were kept as batch cultures in their respective condition for over 1 year before the start of the experiment. Iron addition was done using an acidified (pH = 2) 10 mM solution of FeCl_3_ to reach a 10 μM final concentration of FeCl_3_. Residual iron in the low-iron treatment was sufficient to support a slow growth rate for *T*. *oceanica* maintained by biweekly transfers. The general work was done in a trace-metal clean environment. Divalent trace metals were removed from the ASW by gravity flow-through a Sigma Fritted Luer Lock Chromatography Column (2.5cm x 23cm; Sigma-Aldrich Corporation, St. Louis, Missouri, USA) filled with 100 mg Chelex^®^100 (BioRad, Hercules, CA, USA) in a trace metal clean working space and a speed of 1–2 drops per second.

### Experimental design

The experimental design was followed as described elsewhere [33]. Briefly, *Thalassiosira oceanica* cultures were kept in exponential growth, and sampling was done in low- to mid-exponential phase. A 22 h long (long-term (LT)) and a 6 h long (short-term (ST)) experiment were performed separately. The LT-experiment included high-iron, low-iron, and iron-recovery cultures. While high-iron cultures were grown with a final concentration of 10 μM FeCl_3_, no iron was added to the low-iron cultures. Ten μM FeCL_3_ was introduced to the iron-recovery samples after the initial measurement (T_0_). The ST-experiment included high-iron, low-iron, and iron-recovery, as described above, and, additionally, treatments with actinomycin D and iron (actd-Fe), only actinomycin D (actD), DMSO with iron (DMSO-Fe), and DMSO without iron (DMSO). All four treatments were prepared in 500 ml polycarbonate (PC) bottles, with 300 ml for the actD treatments and 200 ml for the DMSO treatments. Cultures in the LT-experiment were grown in 4 L polycarbonate bottles for the sampling of high- and low-iron treatments, and two 4 L bottles were combined for the iron-recovery treatment. For the ST-experiment, 2 L polycarbonate bottles were used. A cell density of 2000 cells/ml was used for the LT-experiment, while the inoculum in the ST-experiment was increased to 75000 cells/ml. The sampling times are shown in Fig 1. All treatments in each experiment were performed as triplicates and only actD and actD-Fe in the ST-experiment were run as duplicate experiments.

### Actinomycin D and DMSO treatment

Actinomycin D (actD) (Invitrogen, Carlsbad, CA, USA) treatments were done with a final concentration of 10 μg/ml actD in DMSO. DMSO concentration in the media was 0.1% final.

### Flow cytometry

The Accuri C6 (Becton, Dickinson and Company (BD), Franklin Lakes, New Jersey, USA) was used to measure cell counts and to gain information about granularity using side-scatter (SSC), cell size using forward scatter (FSC), and chlorophyll content using red fluorescence measurement (FL3, > 670 nm LP; Excitation wavelength 488 nm). The FL3 channel was also used to gate the *T*. *oceanica* population. The *T*. *oceanica* population was used to calculate the growth rate as doubling time per 24 h using, using μ = ((ln N_t_ − ln N_0_)/t) x 24. N_t_ is the cell number at timepoint 1, and N_0_ is the cell number at timpoint 0. All measurements were done on a 96well plate using 200 μl of culture. Samples were measured as duplicates in the ST-experiment and as triplicates in the LT-experiment.

### Chlorophyll measurements

Ten ml culture was filtered onto glass fibre filters (GFF) and stored in scintillation vials at -20°C until further processing. Chlorophyll was extracted in 90% acetone at -20°C for 24 h. The measurement and the calculation were done using the Welschmeyer method [62] with a 10-005R fluorometer (Turner Designs, San Jose, CA, USA).

### POC/PON measurements

Twenty ml of culture was used for the analysis of particulate organic carbon (POC) and particulate organic nitrogen (PON). The samples were filtered onto combusted GFF filters, which were dried for 24 h at 52°C following filtering. GFF filters were packed in tin cups and combusted at 1000°C to measure total organic C and N on an Elementar VarioMicro Cube Elemental Analyzer (Elementar, Langenselbold, Germany).

### DUAL-PAM-100 measurements

A Waltz DUAL-PAM-100 (Heinz Walz GmbH, Effeltrich, Germany) was used to measure photo physiological parameters, including normalized variable fluorescence (F_v_/F_m_) for LT and ST-experiment. Additionally, electron transfer rate (ETR), effective photochemical quantum yield (Y(II)), non-photochemical quenching (Y(NPQ)), and non-regulated heat dissipation (Y(NO)) were measured during the ST-experiment using light curve measurements, including 11 steps saturation pulse with increasing light intensity (0; 15;22;31;62;104;135;225;348;540;834 μE). Y(II), Y(NPQ), and Y(NO) are complimentary PS II quantum yields, describing the partitioning of absorbed excitation energy [63,64]. All measurements were done following a 10 min dark incubation with default settings for saturation pulse and measuring light (SP = 10.000 μE for 600 ms, ML = 128 μE) in the LT-experiment. Optimization of the light curve for F_v_/F_m_ measurements led to an adjustment for the ST-experiment, resulting in a saturation pulse of level 1 (1000 μE for 600 ms) with a measuring light of level 10 (128 μE).

### RNA extraction

RNA extraction was done with the Qiagen Plant RNeasy kit (Qiagen, Inc., Valencia, CA, USA) using a 150–200 ml sample following the manufacture’s protocol. The extraction was done in 450 μl RLC buffer using a 5 min incubation at 56°C without sonification. Samples from the LT samples were filtered onto 2 μm PC filters, washed off, and pelleted by centrifugation for 3 min at 5000 g before flash-frozen in liquid nitrogen. The samples from the ST-experiment samples were filtered on 2 μm PC filters and immediately flash-frozen in liquid nitrogen. All samples were stored at– 80°C. RNase-free DNase by Qiagen (Qiagen, Inc., Valencia, CA, USA) was used for on-column DNA digestion. RNA quantification was done with a Nanodrop (Thermo Fisher Scientific, Waltham, Massachusetts, USA).

### Transcript analysis using NanoString

The probes for the NanoString (NanoString Technologies ^®^, Seattle, Washington, USA) analysis were designed based on the genome of *T*. *oceanica* [14] (Accession nr. AGNL01000000). The cross-binding activity was checked with a blast approach using Needle Wunsch scores (S5 Fig). The first set of probes included a total of 44 mRNA probes which were specifically designed for the LT-experiment (S2 Table). After the LT-experiment was conducted and measured, the short-term experiment was developed, and 24 probes were designed to analyze the samples of the ST-experiment and the LT-experiment in one combined targeted transcript analysis (S2 Table).

The first probe set (44 mRNA probes) was used in the first analysis, the long-term experiment. Here, the mRNA transcript levels were measured using 100 ng RNA (LT-Run) (S2 Table). The hybridization time was set at 20 h for the probes to bind the targeted transcripts in our samples. The “nCounter^®^ XT assay” protocol from NanoString was followed.

In the second analysis, using the 24 mRNA probe set, samples from LT- and ST-experiments were analyzed combined in two 96well plates (96well-Run) (S2 and S3 Tables). The “nCounter® PlexSet™ Reagents for Gene Expression User Manual” was followed. Each column on the 96well plate was pooled after the 20 h hybridization incubation to create one sample that was processed and analyzed. Two separate probes were used, Probe A and Probe B (S2 Table). Probe A has a target-specific sequence and a row-specific fluorescently labelled reporter tag, and Probe B has the universal capture tag. Based on high counts in the first LT-run, FLDA1, FBA3, FBA4, and PETE were attenuated by the addition of inactive probes, aiming for a 90% reduction of probe counts. A titration run was performed before the 96well plate run on a low-iron, high-iron, and iron-recovery sample to ensure sufficient counts of targets. The titration run was used to calculate loading amounts of the different treatments, as well as the actual attenuation factor, which was used for the final analysis of the 96well plates (see below). Based on the titration run, 90 ng of RNA was used for high-iron samples (including iron-recovery samples sampled later than 30 min after the addition of iron), 70 ng for low-iron samples, and 80 ng for iron-recovery samples. All low-iron samples and iron-recovery samples within 30 min after iron addition were attenuated for the genes mentioned earlier.

### Data analysis

#### The standard calculation of transcript counts

The generated counts for each transcript were downloaded and processed in multiple steps, starting in the NSolver software. The geometric mean of the Top 3 positive control probes (included in the probeset by NanoString) was used to normalize the efficiency of the hybridization reaction. Next, the arithmetic mean over all samples of one house-keeping gene, the nuclear-import-exporter (THAOC_05312), was used to correct loading differences. This gene revealed even expression patterns in our study (S9 Fig). Other potential house-keeping genes showed strong significant differences between nighttime and daytime samples and were excluded from the calibration (S9 Fig).

The 96well-plate run included a reference lane which was run in the first lane on both plates. The reference lane was used for in-plate probe calibration and contained the same sample in each well (Lane1 A-H). Here, a mix of one high-iron and one low-iron sample (1:1) was used to achieve sufficient counts for each target. The calibration of RNA amount loading differences and the correction for the attenuation was done using Excel. The housekeeping gene normalization for the actD samples was done independently, based on the degradation of all transcripts. Therefore, the arithmetic mean of the housekeeping gene counts of each timepoint was used to normalize each timepoint separately.

#### Decay curve analysis and validation

The data points generated from the time-course experiments displayed non-linear trends, and a broken-line model was used to calculate the decay curve. The slope of the curve before it became stable was used for the half-life calculations [65]. The changepoint is the point where the slope suddenly changes.

Decay curve:

$$X_{np}=\beta_{0}\times treatment_{np}+\beta_{1}\times treatment_{np}\times \min\left(time,t_{0}\right)+\beta_{2}\times treatment\times max(time-t_{0,}0)+e$$

$$e∼Normal(0,\sigma^{2})$$

Where Xnp is the log-transformed data with n variables and p samples, and t_0_ is the change point. t_0_ is fixed for each treatment (calculation is shown below). β_0_ is the unknown intercept for each treatment. β_1_ is the unknown slope coefficient of the interaction between treatments before the change point, and β_2_ is the unknown slope coefficients after the change point. Therefore, β_1_ and β_2_ are the decay rate vectors for the different treatments. The calculation of t_0_ for each treatment was done using “lm.br” in R. The “lm.br” function fits the following model to each treatment’s data:

$$y=alpha+B\times \min\left(time-t_{0},0\right)+Bp\times \max\left(time-t_{0},0\right)+e$$

$$e∼Normal(0,\sigma^{2})$$

Y is the log-transformed data for one treatment, while the parameters’ alpha’,’ B’, and’ Bp’ are unknown parameters.

Comparison of decay rates between treatments:

After β1 was calculated, a likelihood ratio test was applied under the following hypothesis:

H_0_: The decay rates are the same between the two treatments.

(eg. β_1__actd = β_1__actd-Fe)

H_a_: The decay rates are different between the two treatments.

(eg. β_1__actd ≠ β_1__actd-Fe)

The P-value of the test showed how strong the evidence was to reject that two treatments share the same decay rates. The decay rate was used to calculate the half-life by t_1/2_ = ln2/k, with k being the decay rate.

#### Visualization of transcript counts

The normalized transcript counts were analyzed and visualized in R [66] using ggplot2 [67]. The visualization included the plotting of transcript counts over time to visualize changes. Fold changes between high-iron and low-iron samples were calculated, and a two-tail Student’s T-test (with unequal variance) was used for significance tests (p <0.05).

#### Correlation matrix analysis and principle component analysis

The correlation matrix analysis based on Spearman correlation was generated using log2 values of all transcript counts, excluding samples that were treated with actinomycin D, using the R-package ComplexHeatmap [68], belonging to the Bioconductor package [69]. The final curation of the heat map was done with Adobe Illustrator. The principle component analysis was done in R using the “prcomp” function. Illustration in R was done with autoplot and refined in Adobe Illustrator.

### In silico analysis

The *in-silico* analysis of putative transcription factors and conserved domains in CREGx2 was performed using the Conserved Domain search tool from NCBI [70] on all proteins from *T*. *oceanica* [71] using default settings. The following accession numbers of superfamilies were found: HSF—cl12113; Myp—cl21498; bzip—cl21462; zf-C2H2—cl20464, cl16448, cl22457, cl16472, cl05634; zf-CCHC—cl11592, cl20584, cl22700; zf-TAZ—cl02660; bHLH—cl16716, cl13910, TRF—cd11660; homeobox—cl0008; AP2—cl00033; TAF—cl05005, cl08418, cl04653; E2F - cl03534; CBF—cl20259, cl00074; Sigma70—cl22432, cl02812, cl08419. An NCBI blast analysis (blastp) was used to confirm putative transcription factors. In general, *in-silico* analysis of protein localization was done with TargetP and ChloroP [58,59]. The NetNGlyc 1.0 Server was used for the prediction of N-glycosylation sites in CREGx2 [72], and the SignalP 4.0 Server [73] was used for signal peptide prediction. The maximum likelihood tree of CREGx2 (THAOC_08512) was done in MEGA [44] using the top 100 blastp hits. The sequence alignment was done using a muscle alignment, and a maximum likelihood tree was generated with the WAG model and gamma distribution. The tree was further edited in itol [74] and Adobe illustrator. Conserved domain search in CREGx2 and its related proteins was done using the Conserved domain search tool in NCBI [70], and the ScanProsite server [75] was used for histidine-rich site prediction.

## Supporting information

S1 Fig Changes in cell properties based on flow cytometry data. Box plots (A-C) and short and long time-courses (D-F) of FL3 (B, F), SSC (C, G) and FSC (D, H). High-iron, low-iron, and iron-recovery samples are shown in black, blue, and orange, respectively. A two-tailed Student’s t-test was used for the statistical analysis of box plots (A-C). Box plots represent duplicate measurements of three experiments (n = 6) for each time point (0 h, 1 h, 6 h). Line graphs (D-F) show the trend of each parameter over the 22 h sampling period divided into long-term and short-term experiments. Error bars represent standard errors (S.E.). Statistically significant P values are indicated as * <0.05, **, 0.01, *** < 0.001, **** < 0.0001. A line without stars indicates a test that is not statistically significant.
(TIF)

S2 Fig Comparison of flow cytometric parameters in low-iron, high-iron, and iron-recovery samples. Characterization of cells grown under low-iron, high-iron, and iron-recovery conditions. High-iron samples are black, low-iron samples are blue, and iron-recovery samples are shown in orange. Results from ST- and the LT-experiment are combined, with every dot representing one sample. We used a two-tailed Student’s T-test for statistical analysis between high-iron and low-iron cultures. Statistically significant P values are indicated as * <0.05, ** < 0.01, *** < 0.001, **** < 0.0001. A line without stars indicates a test that is not statistically significant. (A) FL3 (LP > 670 nm) measurements (relative units) (B) Side scatter (SSC) measurements (relative units). (C) Forward scatter (FSC) measurements (relative units).
(TIF)

S3 Fig Overview of fold changes between high- and low-iron samples. (A) All genes analyzed in the ST and the LT-experiment are shown with their mean counts across all high- and low-iron samples on the x-axes and their fold change between high- and low-iron on the y-axes. The results from both experiments, the ST- and the LT-experiment, are combined in this analysis. All values are log2-transformed for better comparison. (B) The seven transcripts with the highest fold changes. Box plots representing transcript levels high- and low-iron conditions are plotted with Student’s t-test used for significance analysis between high- and low-iron transcript counts. Statistically significant P values are indicated as * <0.05, ** < 0.01, *** < 0.001, **** < 0.0001. A line without stars indicates a test that is not statistically significant.
(TIF)

S4 Fig Transcript counts of the first LT run over the period of 22 h. Normalized transcript count results from the first NanoString run of 44 targeted transcripts. The x-axes are in hours, and three treatments are shown. High-iron samples are black, low-iron samples are blue, and iron-recovery samples are orange. The addition of 10 μM FeCl_3_ was done after the initial measurement at timepoint T_0._
(TIF)

S5 Fig Percent identity of all probe sequences aligned to each gene. All target sequences were divided into their respective probe A and probe B, resulting in 50bp length. Each probe was then aligned with all targeted genes that were analyzed in this study. The alignment was done using the Needleman-Wunsch algorithm with a gapopen penalty of 99 and a gapextend penalty of 10 (max), aiming to align the probes without gaps. X-axes are percent identity, and y-axes are Needleman-Wunsch scores. The size of each circle represents the number of alignments included in this circle. The exact hit between the probe and its target gene has 100% identity and a Needleman-Wunsch score of 250. The sequences higher than the 75% identity threshold show very low Needleman-Wunsch scores, which indicated partial alignment of the probes.
(TIF)

S6 Fig Correlation analysis of ISIP1/ISIP3 and NanoString probe analysis for putative cross binding. (A) All transcripts counts (log2 values) from ISIP1 and ISIP3 are plotted to show their correlation. ActD (red), DMSO (purple), high-iron (grey), low-iron (blue), iron-recovery (orange). (B) Alignments of ISIP1 probe A and probe B onto full-length ISIP1 are shown. Dark blue indicates matching nucleotides. The percent identity is shown to the right of the alignments. A 75% identity is needed for possible false binding [56]. (C) Alignment of ISIP1 Probe A and B onto full-length ISIP3 is shown. Dark blue indicates identical nucleotides in both sequences, with the full alignment percent identity shown to the right of each alignment.
(TIF)

S7 Fig Correlation analysis of FBA3/FBA4 and NanoString probe alignment for possible cross binding activity. (A) Correlation analysis of all sample points for FBA3 and FBA4. actD (red), DMSO (purple), high-iron (grey), low-iron (blue), iron-recovery (orange). (B) Alignments of FBA3 probe A and probe B onto full-length FBA4 are shown. Dark blue indicates matching nucleotides. The percent identity is shown to the right of the alignments. 75% identity is the threshold for possible false binding (Kane *et al*., 2000). (C) Alignment of FBA4 Probe A and B onto full-length FBA3 is shown. Dark blue indicates matching nucleotides, and the percent identity of the alignment is shown to the right.
(TIF)

S8 Fig Heatmap clusters 1 and 3 with sample-specific names. Clusters 1 and 3, as outlined in the text and indicated in Fig 8, are shown with sample-specific names. The name is divided into treatment (high/low), experiment-type (LT-long-term experiment), and the point of time after iron addition as experiment-specific identifiers. Twelve h and 14 h are 7 PM and 9 PM, respectively.
(TIF)

S9 Fig Transcript counts of potential housekeeping genes. (A) Transcript counts of potential housekeeping genes, including the total amount of transcript reads over all transcripts (sum). The significant difference between low- and high-iron samples in the 24_probeset for the nuclear import-export protein is based on loading issues. The sum of all transcripts over all genes (sum) reveals the same trend indicating that the loading amounts were higher than expected. (B) Box plots representing transcript levels of high-. low-iron, and iron-recovery samples are plotted with Student’s t-test used for significance analysis. (C) Box plots of transcript counts of daytime and nighttime samples. These samples are further divided into low-iron, high-iron, and recovery samples from the 24_probeset NanoString analysis. Daytime boxplots are shown in orange, and nighttime boxplots are presented in blue. (D) Box plots of transcript counts of daytime and nighttime samples. The samples are further divided into low-iron, high-iron, recovery samples from the 48_probeset NanoString analysis. Day time boxplots are shown in orange and nighttime samples are in blue. Overall, the top row shows results from DNAj, the second row shows the nuclear import-export protein, and the third row is a hypothetical transporter protein. The sum of all genes is the bottom row. High iron samples are shown in black, low iron samples are shown in blue and recovery samples are shown in orange. Statistically significant P values are indicated as * <0.05, ** < 0.01, *** < 0.001, **** < 0.0001. A line without stars indicates a test that is not statistically significant.
(TIF)

S1 Table THAOC numbers of all targeted transcripts. This list includes the identifiers of the targeted transcriptome experiment. The probes were designed based on these sequences.
(XLSX)

S2 Table List of target sequences for NanoString probes. The table provides an overview of all NanoString probes. It shows the 100 bp long target sequences that were used for the design of the NanoString probes in the LT-experiment. Additionally, the table shows the sequences of probe A and probe B used in the ST-experiments. Probe A includes 50 nt of the target sequence and the sequence that attaches to the row-specific fluorophore. The sequences for probe B include 50 bp of the target sequence and a short universal sequence for the capture probe (CGAAAGCCATGACCTCCGATCACTC).
(XLSX)

S3 Table Overview of Plate 1 and Plate 2 of the 96well plate run for the targeted transcriptome analysis. Attenuated samples are shown in bold. The first lane is a reference lane, loaded with the same sample mix (high:low; 1:1). The sample name consists of the type: Low (no iron added), Rec (Iron added after initial measurement), high (grown with 10 μM FeCL_3_), ActD (addition of actinomycin D after the initial measurement), ActD+Fe (addition of actD and iron after the initial measurement), DMSO (addition of DMSO after initial measurement), DMSO+Fe (addition of DMSO and iron after the initial measurement). This is followed by the time (0 h for the initial measurement) taken after the addition of iron in min (‘)/ hours (h) and a unique experiment identifier.
(XLSX)

S4 Table Changepoints and half-lives in min. Based on the transcripts counts following the addition of iron (rec) or the addition of actinomycin D (actD) or actinomycin D and iron (actDFe) a changepoint was determined. The changepoint is the point of time when the slope reaches its plateau. The half-life was calculated based on the slope of the curve before reaching the changepoint used as k in t_1/2_ = ln2/k.
(XLSX)

S5 Table Overview of calculated changepoints and the p-value for targeted transcripts. The table shows the changepoints (CP) of targeted transcripts for three different treatments and the significance of the slope difference between and after the changepoint (p < 0.01).
(XLSX)

S1 File Raw and normalized transcript counts. The four tables show the raw and normalized transcript counts from the targeted transcript analysis divided into the 44_probeset and the 24_probeset.
(XLSX)
