# Supplementary figures and images for: Short-term response to iron resupply in an iron-limited open ocean diatom reveals rapid decay of iron-responsive transcripts
Source: PLoS One. 2023 Jan 24;18(1):e0280827. doi: 10.1371/journal.pone.0280827 (PMC9873189; doi:10.1371/journal.pone.0280827)

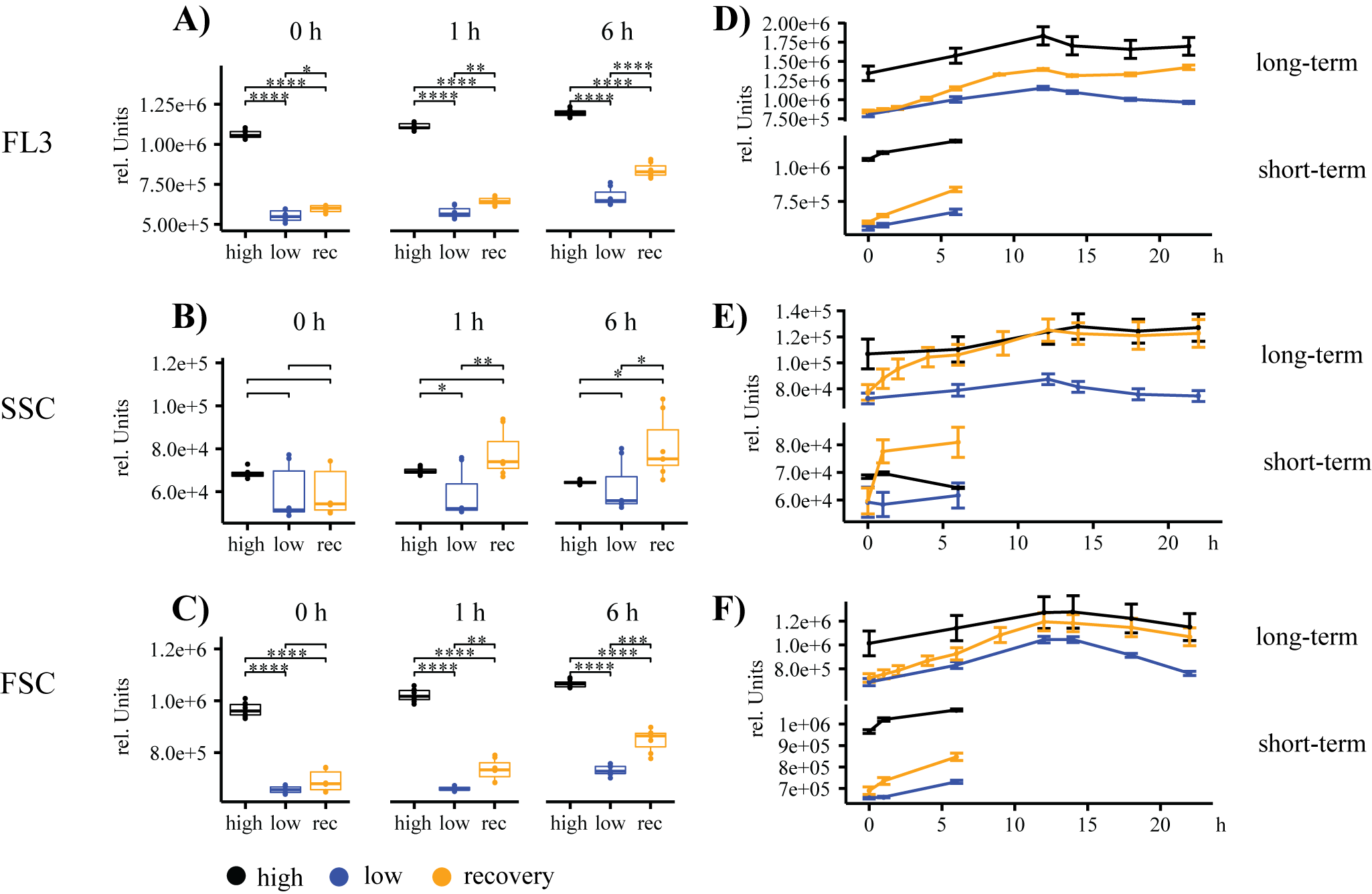

Supplement: S1 Fig — Box plots (A-C) and short and long time-courses (D-F) of FL3 (B, F), SSC (C, G) and FSC (D, H). High-iron, low-iron, and iron-recovery samples are shown in black, blue, and orange, respectively. A two-tailed Student’s t-test was used for the statistical analysis of box plots (A-C). Box plots represent duplicate measurements of three experiments (n = 6) for each time point (0 h, 1 h, 6 h). Line graphs (D-F) show the trend of each parameter over the 22 h sampling period divided into long-term and short-term experiments. Error bars represent standard errors (S.E.). Statistically significant P values are indicated as * <0.05, **, 0.01, *** < 0.001, **** < 0.0001. A line without stars indicates a test that is not statistically significant. (TIF) [file pone.0280827.s001.tif]

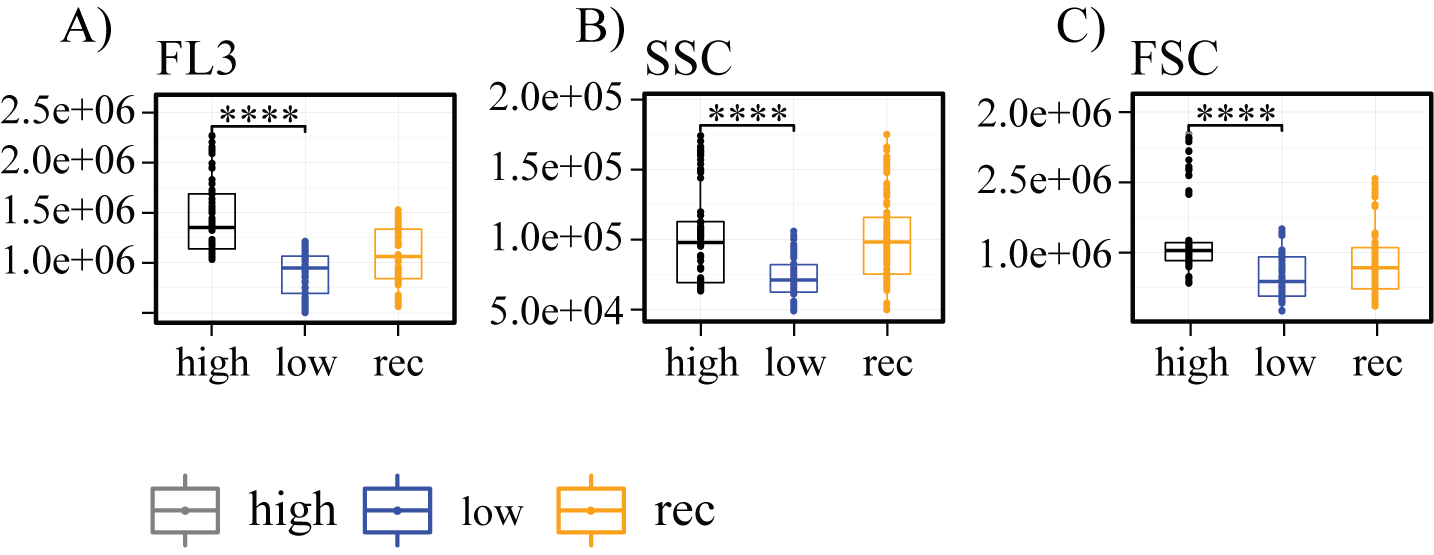

Supplement: S2 Fig — Characterization of cells grown under low-iron, high-iron, and iron-recovery conditions. High-iron samples are black, low-iron samples are blue, and iron-recovery samples are shown in orange. Results from ST- and the LT-experiment are combined, with every dot representing one sample. We used a two-tailed Student’s T-test for statistical analysis between high-iron and low-iron cultures. Statistically significant P values are indicated as * <0.05, ** < 0.01, *** < 0.001, **** < 0.0001. A line without stars indicates a test that is not statistically significant. (A) FL3 (LP > 670 nm) measurements (relative units) (B) Side scatter (SSC) measurements (relative units). (C) Forward scatter (FSC) measurements (relative units). (TIF) [file pone.0280827.s002.tif]

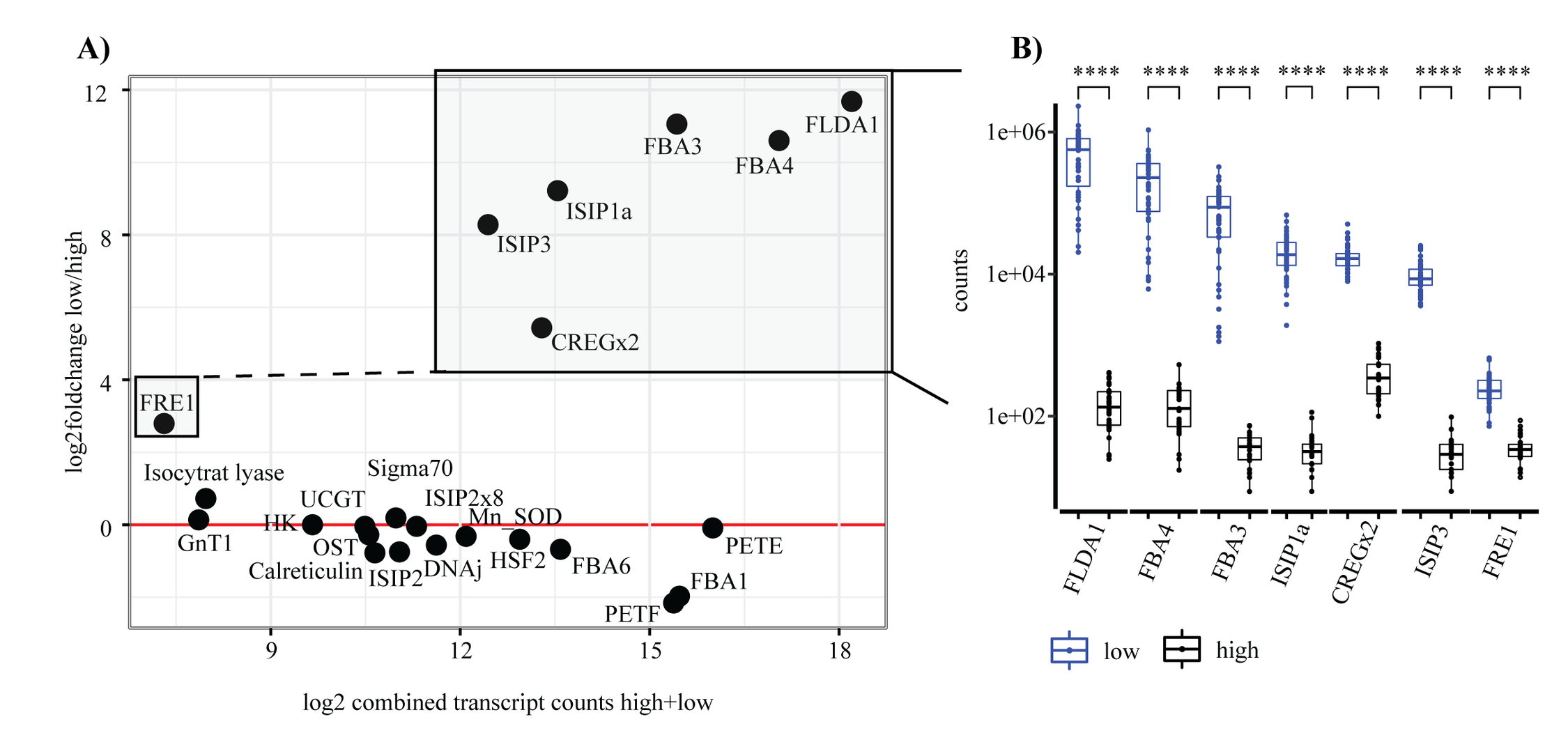

Supplement: S3 Fig — (A) All genes analyzed in the ST and the LT-experiment are shown with their mean counts across all high- and low-iron samples on the x-axes and their fold change between high- and low-iron on the y-axes. The results from both experiments, the ST- and the LT-experiment, are combined in this analysis. All values are log2-transformed for better comparison. (B) The seven transcripts with the highest fold changes. Box plots representing transcript levels high- and low-iron conditions are plotted with Student’s t-test used for significance analysis between high- and low-iron transcript counts. Statistically significant P values are indicated as * <0.05, ** < 0.01, *** < 0.001, **** < 0.0001. A line without stars indicates a test that is not statistically significant. (TIF) [file pone.0280827.s003.tif]

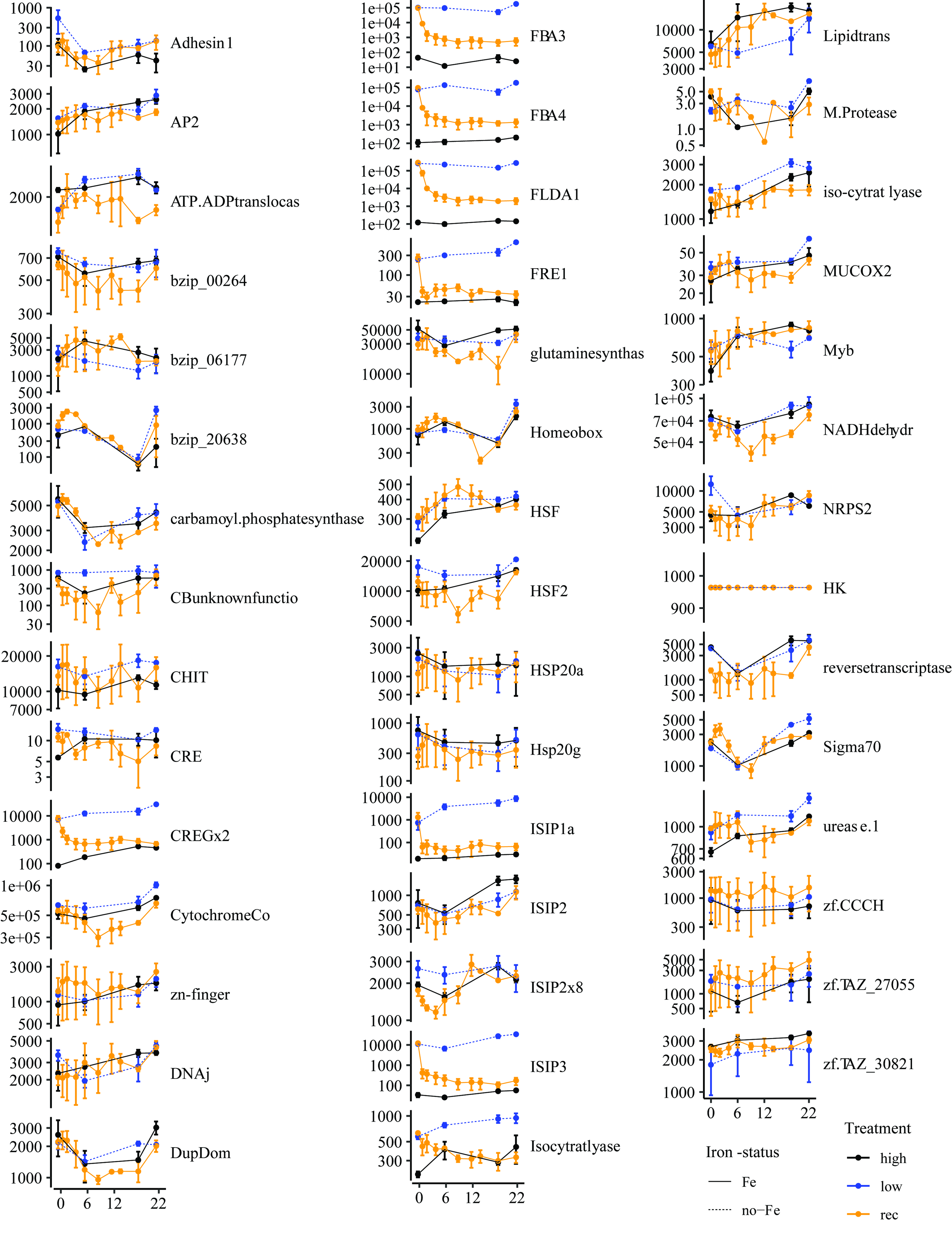

Supplement: S4 Fig — Normalized transcript count results from the first NanoString run of 44 targeted transcripts. The x-axes are in hours, and three treatments are shown. High-iron samples are black, low-iron samples are blue, and iron-recovery samples are orange. The addition of 10 μM FeCl3 was done after the initial measurement at timepoint T0. (TIF) [file pone.0280827.s004.tif]

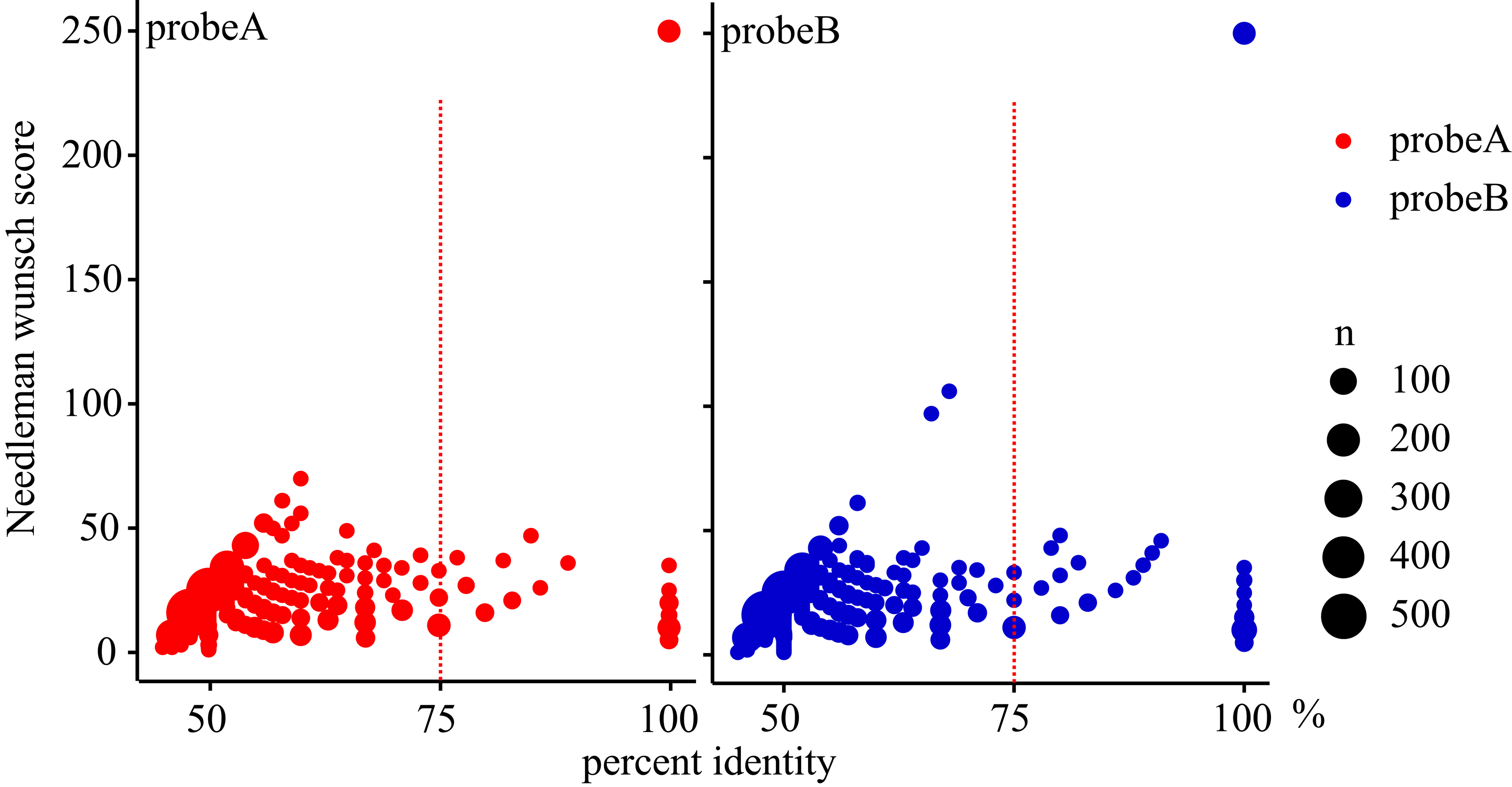

Supplement: S5 Fig — All target sequences were divided into their respective probe A and probe B, resulting in 50bp length. Each probe was then aligned with all targeted genes that were analyzed in this study. The alignment was done using the Needleman-Wunsch algorithm with a gapopen penalty of 99 and a gapextend penalty of 10 (max), aiming to align the probes without gaps. X-axes are percent identity, and y-axes are Needleman-Wunsch scores. The size of each circle represents the number of alignments included in this circle. The exact hit between the probe and its target gene has 100% identity and a Needleman-Wunsch score of 250. The sequences higher than the 75% identity threshold show very low Needleman-Wunsch scores, which indicated partial alignment of the probes. (TIF) [file pone.0280827.s005.tif]

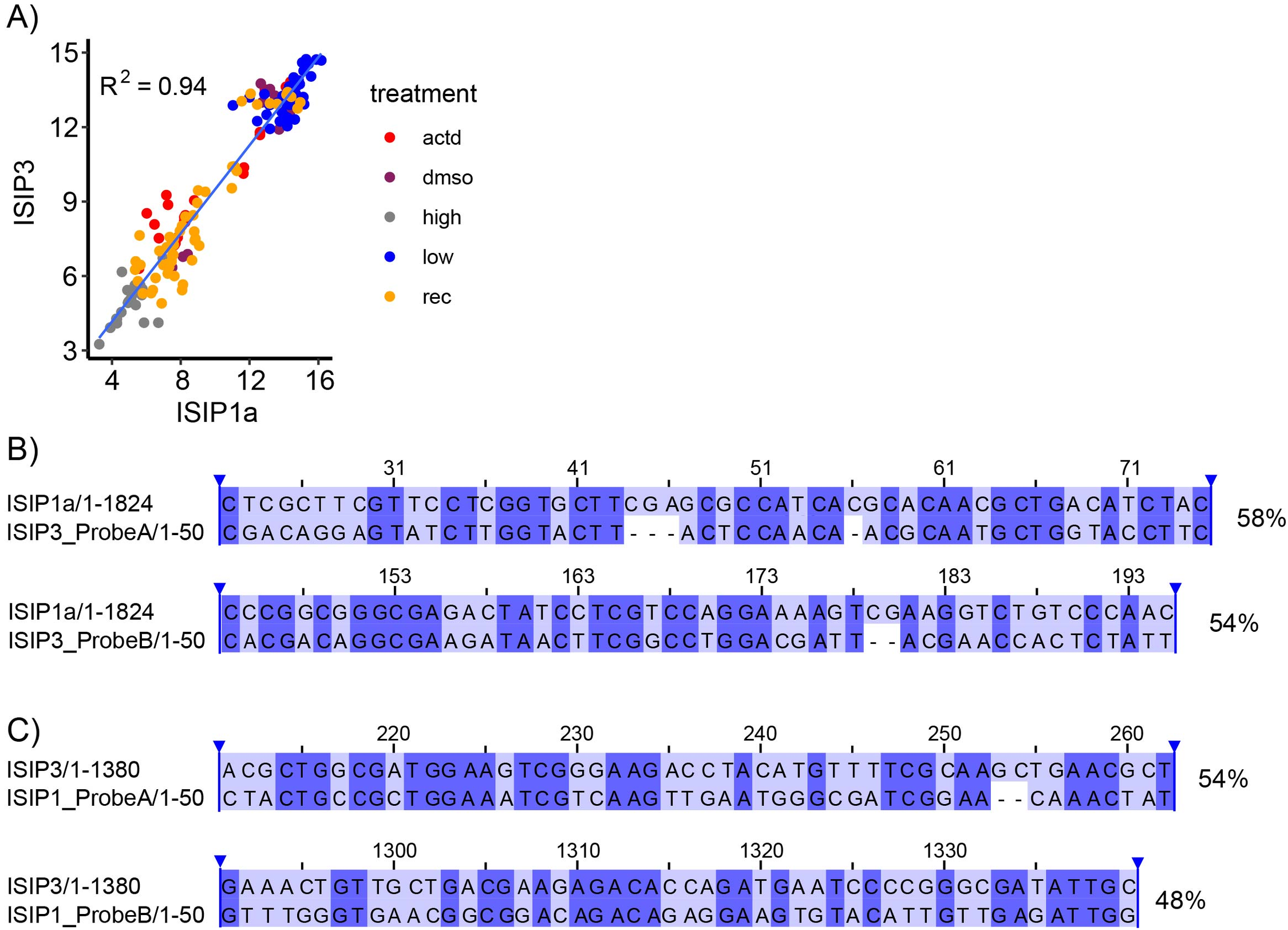

Supplement: S6 Fig — (A) All transcripts counts (log2 values) from ISIP1 and ISIP3 are plotted to show their correlation. ActD (red), DMSO (purple), high-iron (grey), low-iron (blue), iron-recovery (orange). (B) Alignments of ISIP1 probe A and probe B onto full-length ISIP1 are shown. Dark blue indicates matching nucleotides. The percent identity is shown to the right of the alignments. A 75% identity is needed for possible false binding [56]. (C) Alignment of ISIP1 Probe A and B onto full-length ISIP3 is shown. Dark blue indicates identical nucleotides in both sequences, with the full alignment percent identity shown to the right of each alignment. (TIF) [file pone.0280827.s006.tif]

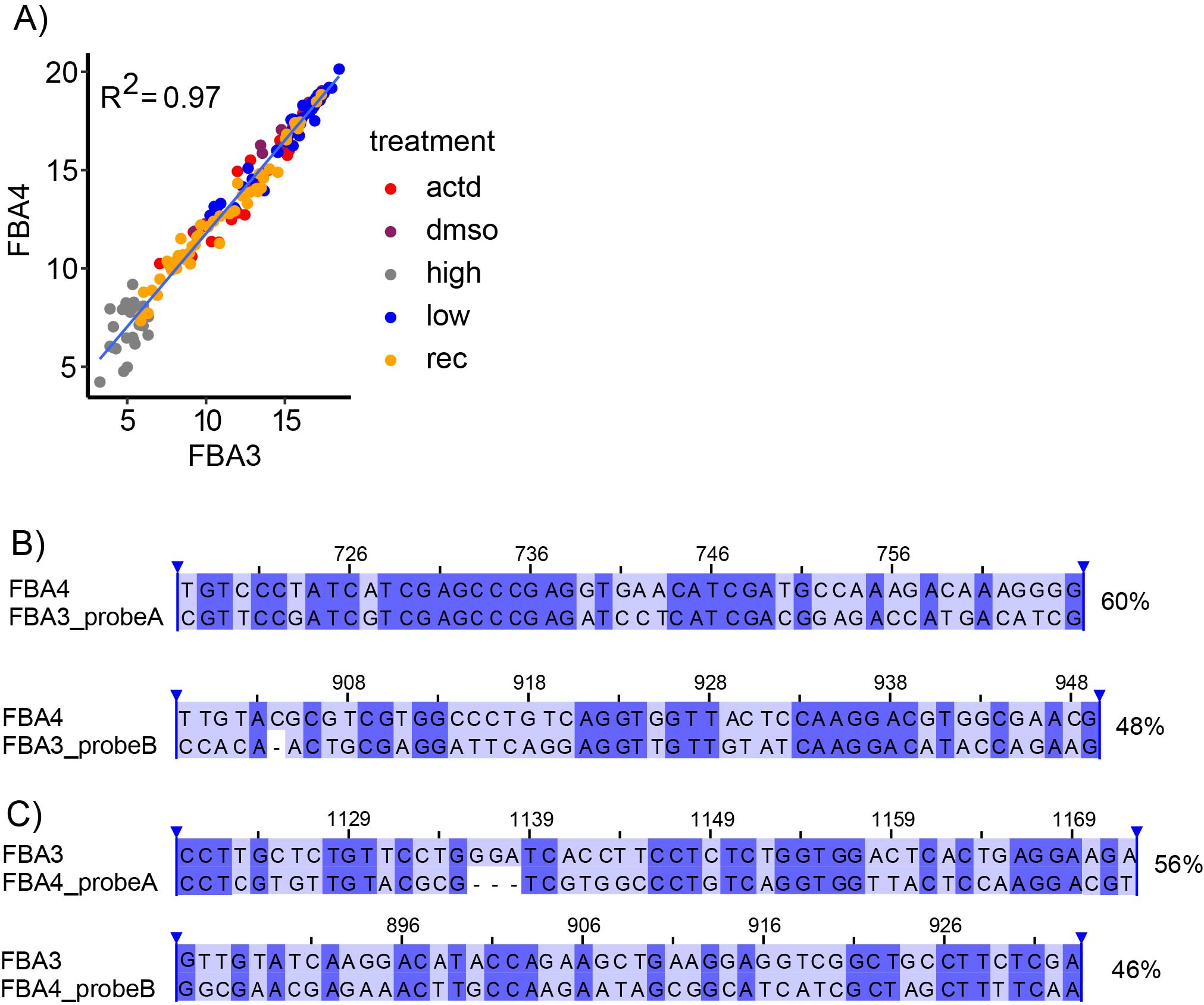

Supplement: S7 Fig — (A) Correlation analysis of all sample points for FBA3 and FBA4. actD (red), DMSO (purple), high-iron (grey), low-iron (blue), iron-recovery (orange). (B) Alignments of FBA3 probe A and probe B onto full-length FBA4 are shown. Dark blue indicates matching nucleotides. The percent identity is shown to the right of the alignments. 75% identity is the threshold for possible false binding (Kane et al., 2000). (C) Alignment of FBA4 Probe A and B onto full-length FBA3 is shown. Dark blue indicates matching nucleotides, and the percent identity of the alignment is shown to the right. (TIF) [file pone.0280827.s007.tif]

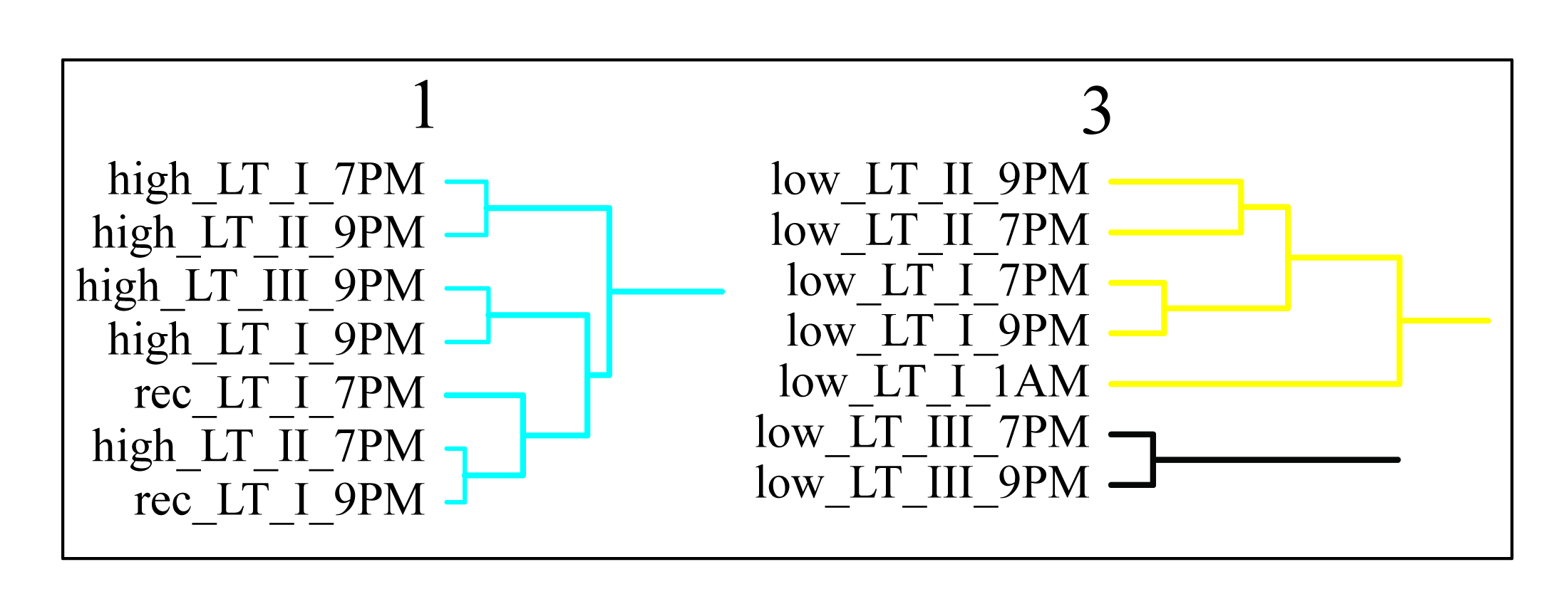

Supplement: S8 Fig — Clusters 1 and 3, as outlined in the text and indicated in Fig 8, are shown with sample-specific names. The name is divided into treatment (high/low), experiment-type (LT-long-term experiment), and the point of time after iron addition as experiment-specific identifiers. Twelve h and 14 h are 7 PM and 9 PM, respectively. (TIF) [file pone.0280827.s008.tif]

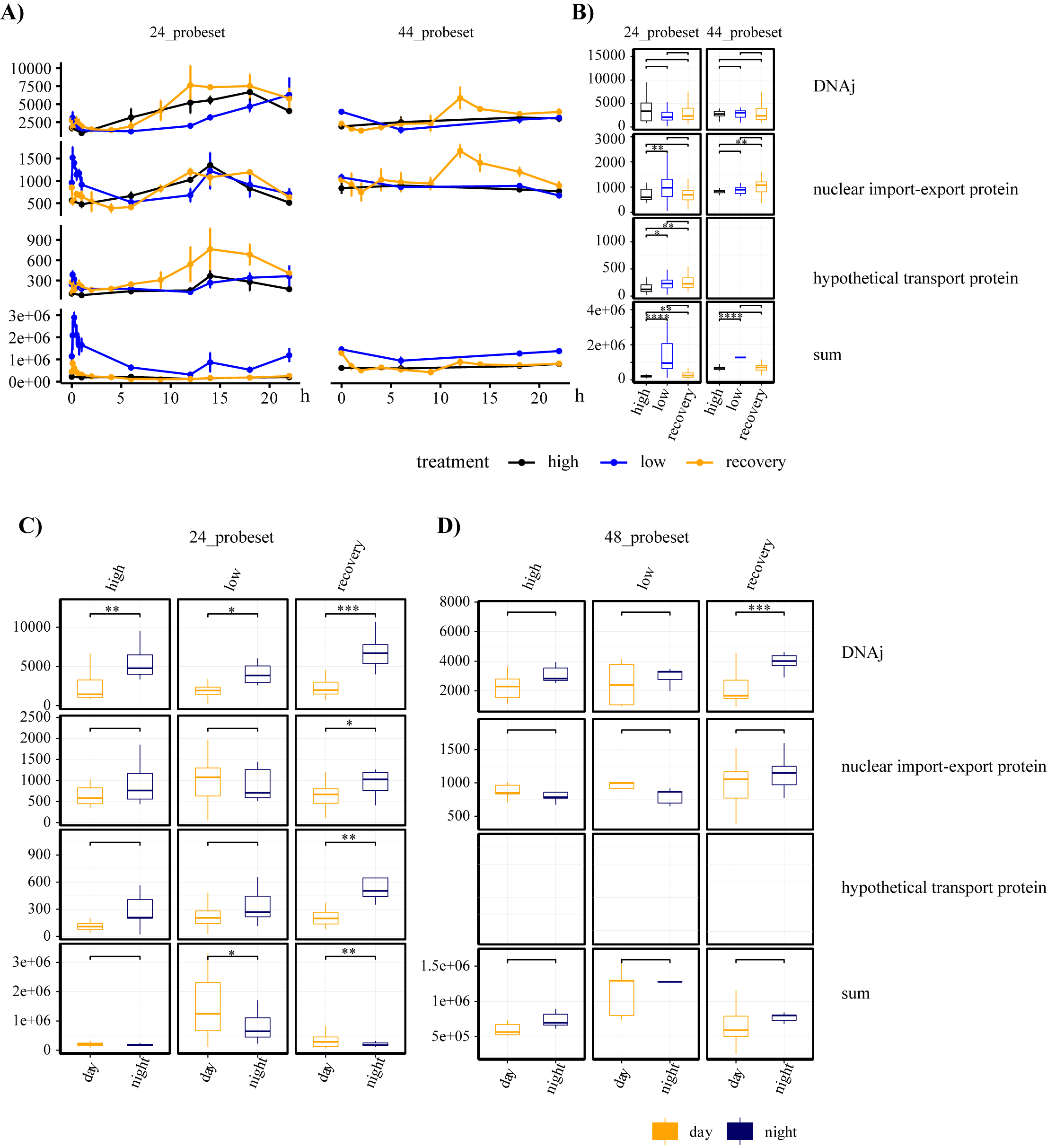

Supplement: S9 Fig — (A) Transcript counts of potential housekeeping genes, including the total amount of transcript reads over all transcripts (sum). The significant difference between low- and high-iron samples in the 24_probeset for the nuclear import-export protein is based on loading issues. The sum of all transcripts over all genes (sum) reveals the same trend indicating that the loading amounts were higher than expected. (B) Box plots representing transcript levels of high-. low-iron, and iron-recovery samples are plotted with Student’s t-test used for significance analysis. (C) Box plots of transcript counts of daytime and nighttime samples. These samples are further divided into low-iron, high-iron, and recovery samples from the 24_probeset NanoString analysis. Daytime boxplots are shown in orange, and nighttime boxplots are presented in blue. (D) Box plots of transcript counts of daytime and nighttime samples. The samples are further divided into low-iron, high-iron, recovery samples from the 48_probeset NanoString analysis. Day time boxplots are shown in orange and nighttime samples are in blue. Overall, the top row shows results from DNAj, the second row shows the nuclear import-export protein, and the third row is a hypothetical transporter protein. The sum of all genes is the bottom row. High iron samples are shown in black, low iron samples are shown in blue and recovery samples are shown in orange. Statistically significant P values are indicated as * <0.05, ** < 0.01, *** < 0.001, **** < 0.0001. A line without stars indicates a test that is not statistically significant. (TIF) [file pone.0280827.s009.tif]
